# Genes and SNPs Involved with Scrotal and Umbilical Hernia in Pigs

**DOI:** 10.3390/genes12020166

**Published:** 2021-01-27

**Authors:** Ariene Fernanda Grando Rodrigues, Adriana Mércia Guaratini Ibelli, Jane de Oliveira Peixoto, Maurício Egídio Cantão, Haniel Cedraz de Oliveira, Igor Ricardo Savoldi, Mayla Regina Souza, Marcos Antônio Zanella Mores, Luis Orlando Duitama Carreño, Mônica Corrêa Ledur

**Affiliations:** 1 Programa de Pós-Graduação em Zootecnia, Departamento de Zootecnia, Centro de Educação Superior do Oeste (CEO), Universidade do Estado de Santa Catarina, UDESC, 89815-630 Chapecó, Brazil; ariene.grando@gmail.com (A.F.G.R.); igorsaavoldii@gmail.com (I.R.S.); mayla.zootecnista@gmail.com (M.R.S.); 2 Embrapa Suínos e Aves, Distrito de Tamanduá, 89715-899 Concórdia, Brazil; adriana.ibelli@embrapa.br (A.M.G.I.); jane.peixoto@embrapa.br (J.d.O.P.); mauricio.cantao@embrapa.br (M.E.C.); marcos.mores@embrapa.br (M.A.Z.M.); 3 Programa de Pós-Graduação em Ciências Veterinárias, Departamento de Ciências Veterinárias, Universidade Estadual do Centro-Oeste, 85015-430 Guarapuava, Brazil; 4 Animal Science Department, Universidade Federal de Viçosa, 36570-900 Viçosa, Brazil; hanielcedraz@gmail.com; 5 Programa de Pós-Graduação em Zootecnia, Departamento de Zootecnia, Universidade Federal do Rio Grande do Sul, UFRGS, 91540-000 Porto Alegre, Brazil; 6 BRF SA, 82305-100 Curitiba, Brazil; luis.carreno@brf-br.com

**Keywords:** gene expression, congenital defects, RNA-Seq, transcriptomics, swine

## Abstract

Hernia is one of the most common defects in pigs. The most prevalent are the scrotal (SH), inguinal (IH) and umbilical (UH) hernias. We compared the inguinal ring transcriptome of normal and SH-affected pigs with the umbilical ring transcriptome of normal and UH-affected pigs to discover genes and pathways involved with the development of both types of hernia. A total of 13,307 transcripts was expressed in the inguinal and 13,302 in the umbilical ring tissues with 94.91% of them present in both tissues. From those, 35 genes were differentially expressed in both groups, participating in 108 biological processes. A total of 67 polymorphisms was identified in the inguinal ring and 76 in the umbilical ring tissue, of which 11 and 14 were novel, respectively. A single nucleotide polymorphism (SNP) with deleterious function was identified in the integrin α M (*ITGAM*) gene. The microtubule associated protein 1 light chain 3 γ (*MAP1LC3C*), vitrin (*VIT*), aggrecan (*ACAN*), alkaline ceramidase 2 (*ACER2*), potassium calcium-activated channel subfamily M α 1 (*KCNMA1*) and synaptopodin 2 (*SYNPO2*) genes are highlighted as candidates to trigger both types of hernia. We generated the first comparative study of the pig umbilical and inguinal ring transcriptomes, contributing to the understanding of the genetic mechanism involved with these two types of hernia in pigs and probably in other mammals.

## 1. Introduction

Pig production is one of the most important livestock activities in the world and its evolution and expansion are mainly due to the development of technologies that combine genetics, management, nutrition and well-being [1], which increase productivity and bring the final product closer to what the consumer idealizes. Meat quality and feed efficiency are traits that have been prioritized by genetic breeding programs [2,3,4]. However, in recent years, studies have also been carried out to improve our knowledge on diseases that persist in production, which bring losses to the entire chain [5,6,7,8,9]. Among these, scrotal (SH)/inguinal (IH) and umbilical hernias (UH) are birth defects often found in pigs [10], causing pain and discomfort to the animals and, consequently, economic losses related to reduced performance [11,12] and increased risk of death [13].

Scrotal hernia is mainly characterized by the displacement of intestinal loops to the scrotal sac, resulting from an abnormality in the inguinal ring [11]. Failure to obliterate the vaginal process [14], impairment of the innervations that act on the site [15] or the failed involution of the internal inguinal ring [16] are processes associated to the manifestation of this defect. Moreover, SH can arise as a result of low resistance of the inguinal region [17] caused by disturbances in the metabolism and hydrolysis of the extracellular matrix components, such as collagen and muscle fiber structures [18], compromising the repair of connective tissue [19]. The incidence of SH in pigs is influenced by genetics and environmental factors. Sevillano et al. [20] have shown that the incidence of SH/IH in Landrace and Large White pigs was 0.34% and 0.42%, respectively.

Umbilical hernia is characterized by the passage of abdominal contents (mainly intestine) to the hernial sac, in the umbilical region [12]. The discomfort and pain can be aggravated when secondary factors are associated with the defect, and the pig welfare is compromised [12]. UH can be related to genetic and non-genetic factors such as navel infections, lesions at the site, obesity and incorrect umbilical cord cutting [21,22]. This defect has been associated with a failure in the process of umbilical ring closure [23,24]. The incidence of UH ranges from 0.55% [25] to 1.5% [23] and it varies according to the management, breed line and production lot [26]. Usually, the UH is not observed at birth, and this defect appears when the pigs are already in the growing period [27]. This demonstrates the difficulty that breeding companies and pig farmers have to eliminate such defect from their herds.

The heritability for SH/IH and UH were estimated in 0.31 [20] and 0.25 [28], respectively, which shows moderate genetic influence in the appearance of these defects. The knowledge of the genetic mechanisms associated with the formation of these anomalies is important to understand their underlying causes, regardless of the type of hernia studied. In pigs, quantitative trait loci (QTL) were detected for the occurrence of SH in pig chromosomes (SSC) 2, 4, 8 (locus SW 933), 13 and 16 [29]. In addition, suggestive QTL for IH and SH were found in seven chromosomes (SSC1, 2, 5, 6, 15, 17 and X) when comparing healthy and herniated pigs [11]. Moreover, candidate genes involved with the manifestation of SH, such as Insulin-like receptor 3 (*INSL3*), Müllerian inhibiting substance (*MIS*) and Calcitonin gene-related peptide (*CGRP*) were also identified [11]. Twenty-two single nucleotide polymorphisms (SNPs) located on chromosomes 1, 2, 4, 10 and 13 in Landrace pigs, and 10 SNPs on SSC 3, 5, 7, 8 and 13 of Large White pigs, were associated with the incidence of SH/IH in these populations [20]. To date, there is a record of 116 QTL/associations related to the appearance of SH/IH in pigs in the Animal QTL database [30].

Regarding UH, there are 55 QTL/associations related to its manifestation in the Pig QTL database (accessed in 17th December 2020) [30]. A SNP in the *CAPN9* gene (Calpain 9) on SSC14 significantly associated with UH has already been detected [31]. In commercial pigs, four candidate genes were identified in QTL regions associated with UH, namely *TBX15* (T-Box 15) and *WARS2* (Tryptophanyl TRNA Synthetase 2, Mitochondrial) in chromosome 4, and *LIPI* (Lipase I) and *RBM11* (RNA Binding Motif Protein 11) in chromosome 13 [32]. A QTL on chromosome 14 of the Landrace breed was highly correlated with UH, where the *LIF* (Leukemia inhibitory factor) and *OSM* (Oncostatin M) genes were identified as candidates for this defect [25].

Another approach that has recently been used to investigate the genetic mechanisms involved with these defects is the whole transcriptome study. Functional candidate genes were prospected for scrotal [6] and umbilical [7] hernias based on differentially expressed (DE) genes between affected and unaffected pigs in the inguinal and umbilical ring tissues, respectively, using RNA sequencing (RNA-Seq). Information about gene expression in those tissues is scarce and there are still many gaps to be elucidated. Interestingly, these previous studies have shown some genes and pathways that seem to be shared between SH and UH, many of them involved with muscle. Therefore, new and systematic analyses on tissues characterization and on common processes involved with both types of hernia are needed.

Although several genetic studies have been performed, no comparison between large-scale gene expression profile of pigs affected with SH from those affected with UH was reported to date. Since SH and UH are related to some extent to muscle dysfunction, our hypothesis is that some muscle-related genes could be involved in the development of both types of hernia. Therefore, the objective of this study was to investigate the common molecular mechanisms and genes involved with these two types of hernia by comparing the SH and UH transcriptomes, as well as to identify and characterize polymorphisms in those transcriptomes.

## 2. Materials and Methods

This study was performed with the approval of the Embrapa Swine and Poultry National Research Center Ethical Committee of Animal Use (CEUA) under the protocol number 011/2014. A diagram summarizing the experiment and the analyses performed can be seen in the Supplementary Materials: Figure S1.

### 2.1. Animals and Sample Collection

**Figure 1:**
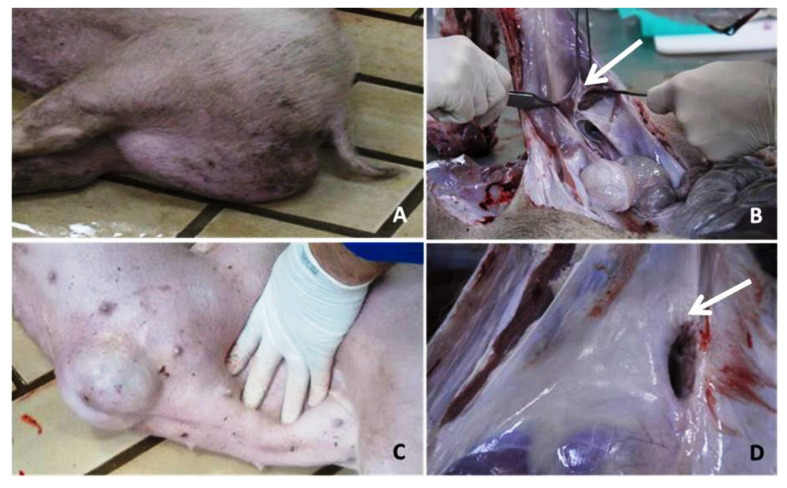
Pathological analysis. Legend: (**A**) swine affected with scrotal hernia. (**B**) Region affected with scrotal hernia (the white arrow shows the inguinal ring). (**C**) Swine affected with umbilical hernia. (**D**) Region affected with umbilical hernia (the white arrow shows the umbilical ring).

Eighteen pigs were selected from a Landrace female line from the same nucleus farm, located in Santa Catarina State, SC, Brazil. From those, five were females affected (case) with UH, five healthy (control) females for UH, four males with SH and four control males for SH, as described by Souza et al. [7] and Romano et al. [6]. Control animals were healthy pigs, without any type of hernia, came from hernia-free litter and were from the same management group of their respective cases. The animals were selected at approximately 60 days of age for SH and 90 days of age for UH. At the Embrapa Swine and Poultry National Research Center, located in Concórdia, SC, Brazil, the pigs were euthanized by electrocution stunning for ten seconds, followed by bleeding, in accordance with the practices recommended by the Ethics Committee. The necropsy was performed for general evaluation and to confirm the both types of hernia. In the pathological analysis, the two groups of animals were confirmed: hernia-affected or without hernias (Figure 1). Tissue samples from the inguinal and umbilical rings were collected for investigating the scrotal and umbilical hernias, respectively (Figure 1). Samples were immediately placed in liquid nitrogen and stored in ultra-freezer (−80 °C) for RNA extraction. Samples from those tissues were also collected and placed in 4% paraformaldehyde for histopathological analysis.

### 2.2. Histopathological Analysis of the Inguinal and Umbilical Ring Tissues

The samples previously collected were routinely processed for histopathology as described by Souza et al. [7]. Briefly, sample tissues were dehydrated in increasing concentrations of ethanol, diaphanized with xylol and embedded in paraffin. Tissue sections were cut with an automatic microtome, stained using the hematoxylin and eosin (HE) and analyzed by optical microscopy.

### 2.3. RNA Extraction, RNA-Seq Libraries Preparation and Sequencing

Total RNA extraction was initiated using the Trizol reagent (Invitrogen, Carlsbac, CA, USA) according to the manufacturer’s instructions. A 100 mg of the tissues were macerated in liquid nitrogen with a mortar and added to a polypropylene tube with 1 mL of the Trizol reagent, mixed using the vortex, and then incubated for five minutes at room temperature (RT, 25 °C). Then, 200 μL of chloroform were added, shaked vigorously and incubated at RT for another five minutes, followed by centrifugation. Approximately 600 μL of the aqueous phase were transferred to a new tube and 600 μL of 70% ethanol were added and homogenized by inversion. This volume was then added to a mini RNeasy silica column (Qiagen, Düsseldorf, Germany) following the manufacturers’ protocol. The quantity and quality of total RNA were assessed by quantification in a Biodrop spectrophotometer (Biodrop, Cambridge, UK), in a 1% agarose gel and in the Bioanalyzer Agilent 2100 (Agilent Technologies, Santa Clara, CA, USA). Samples with an RNA integrity number (RIN) > 8.0 were used to prepare the RNA-Seq libraries.

The RNA of four normal and four SH-inguinal ring and five normal and five UH-umbilical ring tissues were submitted to library preparation with the TruSeq mRNA Stranded Sample Preparation kit (Illumina, Inc., San Diego, CA, USA). For this, 2 μg of total RNA was purified using magnetic microspheres for poly-A selection. The libraries were quantified by qPCR and evaluated in Bioanalyzer (Agilent, Santa Clara, CA, USA), prior to sequencing. Libraries passing the quality control were sent for RNA sequencing in HiSeq 2500 equipment (Illumina, San Diego, CA, USA) at the Functional Genomics Center of ESALQ/USP, in a paired-end protocol (2 × 100 bp). All samples of each hernia and their respective controls were placed in the same lane. Additional information can be found in Romano et al. [6] and Souza et al. [7]. The transcriptome sequences for the scrotal and umbilical hernias used in this study were previously deposited in the SRA database with BioProject numbers PRJNA350530 and PRJNA445856, respectively.

### 2.4. Quality Control and Differentially Expressed Genes

The quality control analysis and mapping were performed using the Bioinformatics Analysis for Quality Control and Mapping (BAQCOM) pipeline available in the Github repository [33]. BAQCOM pipeline uses Trimmomatic tool [34] to remove short reads (<70 bp), reads with low quality (QPhred < 24) and adapter sequences. The sequences were mapped against the swine reference genome (Sus scrofa, assembly 11.1) available on the Ensembl [35] version 95 using the STAR (version 2.7.0) program [36]. To verify the consistency of the expression pattern between the sample groups, principal component analysis (PCA) plots were performed in RStudio [37] (version 1.1.463) from R language [38] (version 3.5.3). The EdgeR package [39] from R was used for the differentially expressed analysis. Differentially expressed (DE) genes in the analyzed tissues (case and control for each hernia) were selected based on the level of false discovery rate (FDR < 0.05) after the Benjamini–Hochberg (BH) method for multiple correction tests [40].

### 2.5. Transcriptomes Characterization of Scrotal and Umbilical Hernia

Initially, SH and UH-related transcriptomes were characterized as the total number of transcripts, number of protein-encoding genes, miRNAs, lncRNAs and non-characterized genes in the swine genome using the Biomart tool available in Ensembl 95 [35]. An annotation of non-characterized genes in the porcine genome was performed using DAVID 6.8 database [41]. The comparison between both transcriptomes was carried out to identify genes expressed in both types of hernia, SH and UH.

### 2.6. Comparison of Differentially Expressed Genes and In Silico Functional Analysis

The classification of DE genes was performed according to the database available in Ensembl 95 and further enrichment in DAVID 6.8 database [41]. A comparison of DE genes between both conditions was performed to verify if the genes were involved in the manifestation of the two types of hernia, and whether there was agreement or not between gene expression profiles in both conditions. In order to verify if DE transcripts named as uncharacterized proteins in Ensembl 95 were similar to genes known in other genomes, the sequences were aligned against the UniProt database [42] using BLASTp tool from the NCBI [43,44]. A gene interaction network was built with the DE genes common to both types of hernia using the STRING database [45,46]. The gene ontology (GO) was evaluated in the Panther [47] and DAVID 6.8 [41] databases, followed by clusterization in the REVIGO tool [48]. Furthermore, it was verified if the DE genes were located in QTL regions previously reported in the Pig QTL database [30].

### 2.7. Identification of Polymorphisms

For the polymorphisms discovery between the transcriptome of animals affected with each type of hernia, the Genome Analysis Tool Kit (GATK) program (version 3.8) [49] was used with the Picard (version 2.5) toolkit [50]. The search for SNPs and insertions or deletions (InDels) was carried out following the filtering parameters and sequence quality suggested by the best practices protocol [51,52]. The polymorphisms annotation for the two hernias studied was performed using the variant effect predictor (VEP) tool (version 3.8) [53] with standard parameters available in the Ensembl 95 database and using the KEGG Pathway database [54]. Therefore, this annotation allowed the discovery of new polymorphisms, as well as to verify their location and possible function in the genome. Using the Biomart data mining tool [35], miRNAs were observed, and a manual comparison was made to identify common miRNAs between SH and UH. Subsequently, a search in the miRBase database (version 22) [55,56] was performed to obtain individual information for each miRNA.

## 3. Results

### 3.1. Histopathological Analysis of the Inguinal and Umbilical Ring Tissues

**Figure 2:**
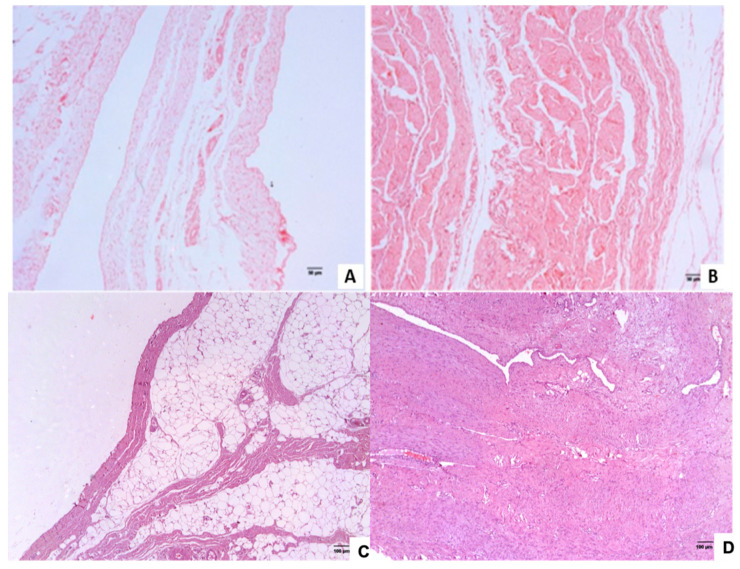
Histopathological slide stained with hematoxylin and eosin (HE). Legend: (**A**) sample from the scrotal hernia (SH)-control group and (**B**) sample from the SH-affected group. A larger number of connective fibers is observed in the sample of the SH-affected group than in the sample from the SH-control group. (**C**) Sample from the umbilical hernia (UH)-control group and (**D**) sample from the UH-affected group. Connective tissue interspersed with adipose tissue is observed in the sample of the UH-control group, while in the sample from the UH-affected group, only proliferated connective tissue is observed.

In the microscopic evaluation, the group affected with SH showed a larger number of connective tissue fibers compared to the control group (Figure 2A,B). In the UH-affected group, the connective tissue was denser than in the control group (Figure 2C,D) as shown by Souza et al. [7].

### 3.2. Sequencing and Mapping

The RNA sequencing of all samples (*n* = 18) generated approximately 465 million paired-end reads and, after the quality control analyses, 13.84% of these were removed, resulting in approximately 400 million reads (86.16%) (Additional file 1: Table S1). The PCA plots show the separation between the affected and control samples from the two evaluated types of hernia (Supplementary Materials 2: Figure S2A–C). Around 93.50% of reads were mapped against the swine reference genome (Sus scrofa 11.1) (Ensembl 95), with individual samples ranging from 87.73% to 96.05%, distributed between the groups of healthy and affected by SH or UH. From those, 78.77% of all reads were mapped in genes. From the 25,880 annotated genes in the swine reference genome (Sus scrofa 11.1, Ensembl 95) [35], 13,307 (51.42%) genes were expressed in the inguinal ring and 13,302 (51.40%) in the umbilical ring.

### 3.3. Characterization of the Scrotal and Umbilical Hernia Transcriptomes

**Figure 3:**
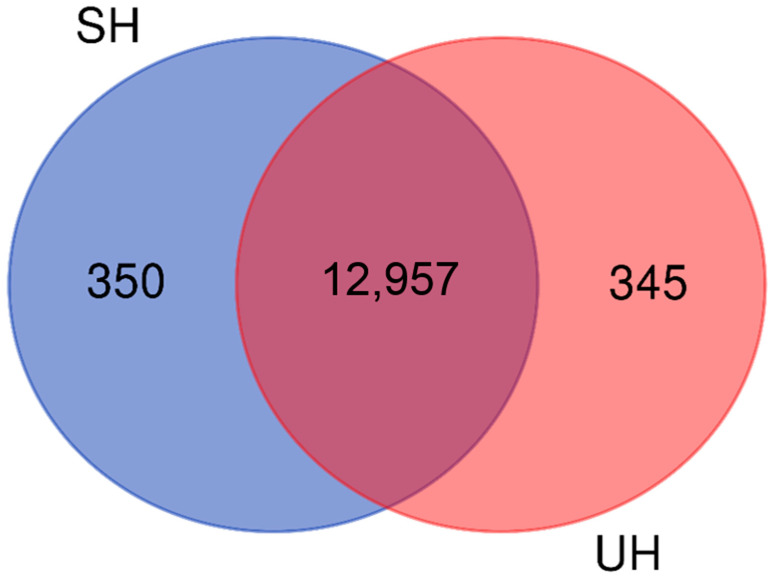
Distribution of transcripts identified in the pig inguinal and umbilical ring tissue samples. Legend: SH—scrotal hernia group; UH—umbilical hernia group. For the SH, the inguinal ring tissue was evaluated, and for the UH, the umbilical ring tissue was analyzed.

The transcripts from the inguinal and the umbilical ring tissues were classified according to the Ensembl 95 database [35] (Table 1). After comparing the two transcriptomes (SH and UH), 94.91% of the genes were identified in both groups (Figure 3). The Venn diagram also presents the number of transcripts expressed exclusively in each type of tissue.

### 3.4. Differentially Expressed Genes

In the pig inguinal ring transcriptome, 627 genes were differentially expressed (FDR < 0.05) between the control and the SH-affected group. Out of those, 435 genes (69.38%) were downregulated and 192 (30.62%) were upregulated in the SH-affected pigs compared to the normal animals. Regarding the genes expressed in the umbilical ring, 199 were DE between normal and UH-affected pigs. From those, 129 were downregulated (64.82%) and 70 (35.18%) upregulated in the UH-affected pigs when compared to the normal ones. In the samples from the SH group, 98.09% of the DE genes were characterized as protein coding genes, 0.64% as lncRNA, 0.32% as pseudogenes, 0.32% as C immunoglobulins, 0.32% as miscRNA, 0.16% as encoding immunoglobulins V and 0.16% as ribozyme. In the UH transcriptome, 92.46% were protein coding genes, 3.52% immunoglobulin C coding genes, 1.51% pseudogenes, 1.01% miscRNAs, 1.01% mitochondrial ribosomal RNA and 0.50% lncRNA.

### 3.5. Differentially Expressed Genes Common to Both SH and UH Transcriptomes

Comparing the DE genes identified in the SH and UH groups, 35 DE genes were present in both transcriptomes (Table 2).

**Figure 4:**
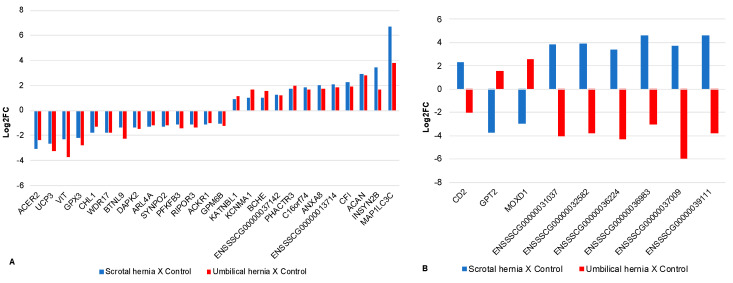
Common differentially expressed genes for scrotal and umbilical hernias and their respective control groups. Legend: (**A**) Genes with similar expression profile and (**B**) with opposite expression profile in the two types of hernia based on the Log2 Fold Change (log2FC).

From the 35 DE genes found in both tissues (inguinal and umbilical ring), 34 were protein coding and one was an immunoglobulin C coding gene. Moreover, eight transcripts (22.86%) were uncharacterized proteins (Table 3), of which six were similar to the amino acid sequences of the pig immunoglobulin and other was similar to another predicted protein in pigs (Table 3). When the relative expression of the 35 common DE genes from each group that represents a type of hernia was compared based on the log2 fold-change (logFC), 26 of these genes had a similar expression profile in the two types of hernia (Figure 4A), and nine had opposite expression profiles considering both types of hernia (Figure 4B).

**Figure 5:**
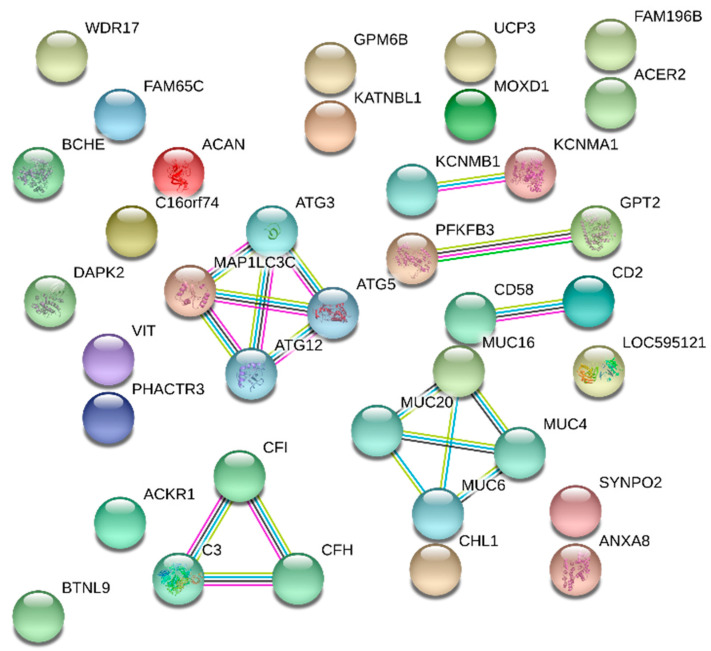
Gene interaction network with differentially expressed genes common to both scrotal and umbilical hernias. Legend: gene network built with 27 of the 35 differentially expressed genes common to both types of hernia obtained with the STRING database using information from *Sus scrofa* proteins.

From the 35 genes DE in both types of hernia, a network with 27 of them was built and the *MAP1LC3C* and *MUC16* genes grouped the two largest clusters of the network (Figure 5). One cluster was related to macroautophagy including the *MAP1LC3C*, *ATG3*, *ATG5* and *ATG12* genes (Figure 5) and the other cluster was composed by the mucin gene family (*MUC4*, *MUC6*, *MUC16* and *MUC20*) (Figure 5), which plays an important role protecting against environmental stress. A third group was related to the complement and coagulation cascade composed of genes *C3*, *CFH* and *CFI* (Figure 5).

Four metabolic pathways were enriched with the 35 genes DE in both types of hernia using the PANTHER database [47]: Huntington’s disease (P00029) (*ARL4A*); muscarinic receptor signaling pathway 1 and 3 of acetylcholine (P00042) (*BCHE*); acetylcholine muscarinic receptor 2 and 4 signaling pathway (P00043) (*BCHE*) and acetylcholine receptor nicotinic signaling pathway (P00044) (*BCHE*). The enrichment of this set of 35 DE genes using the DAVID 6.8 database [41] indicated that those genes participate in 108 biological processes (BP) (Additional file 1: Table S2). The *KCNMA1* gene (potassium calcium-activated channel subfamily M α 1) was the most enriched in BP, appearing in 18 of them (Supplementary Materials 1: Table S2). These BP were clustered in nine macro biological processes (superclusters) using the REVIGO tool [48] (Table 4).

The 26 DE genes with similar expression profile enriched 99 BP (Supplementary Materials 1: Table S3). Considering the molecular function, the set of 35 genes was present in 57 different molecular functions mainly comprising binding, catalytic activity, molecular function regulator, structural molecule activity and transport activity. Using the Pig QTL database [30], two DE genes in both groups of hernias studied here were located in QTL regions already identified as being associated to SH hernia in pigs: the *ACAN* and *BCHE* genes were mapped, respectively, in the QTLs 55892 (SSC7) and 8794 (SSC13).

### 3.6. Identification of Polymorphisms

Using the GATK program with all sequences obtained from the RNA-Seq analyses, 67 polymorphisms were identified in the inguinal ring tissue between SH-affected and unaffected samples (Supplementary Materials 1: Table S4) and 76 in the umbilical ring tissue between UH-affected and unaffected samples (Supplementary Materials 1: Table S5). Comparing the transcriptomes of pigs affected with each type of hernia, the polymorphisms were then classified (Table 5). From the 67 polymorphisms related to scrotal hernia, 56 (83.58%) have already been described in VEP tool and 11 (16.42%) are considered new (Supplementary Materials 1: Table S4). Of the 76 polymorphisms referring to umbilical hernia, 62 (81.58%) have been previously described in VEP tool and 14 (18.42%) are new (Supplementary Materials 1: Table S5).

Considering the whole transcriptome of the two tissues, the variants detected for SH and UH were classified according to the functional region indicating their possible consequences in gene regulation (Table 6). Most of the SNPs in the SH group (37.74%) were classified as synonymous variants (Additional file 1: Table S4), and in the UH group, most were of the UTR3′ type (44.78%) (Supplementary Materials 1: Table S5). In the SH group, two observed variants had calculated SIFT (sorting intolerant from tolerant) score classified as tolerant (SIFT score > 0.05) (Table 7). One of them has already been described in the dbSNP database [35] and the other was classified as new. From the variants belonging to the UH group, six had the calculated tolerance prediction score (SIFT) detected, one of them being deleterious (SIFT ≤ 0.05) and five tolerant (SIFT > 0.05) (Table 7), all of which were already present in the dbSNP database [35]. These six variants belong to six genes, two of which were enriched for metabolic pathways in the KEGG Pathway database [54] (Table 8). The frameshift type variants were located in two genes (*NCOA7* and *SEC62*), of which one was enriched for a metabolic pathway in the same database [54] (Table 8). The SNPs of the SH group were observed in 17 different genes, which enriched nine BP (Table 9) in the DAVID 6.8 database [41]. The SNPs found in the UH group were mapped in 24 genes, which enriched six biological processes (Table 10).

**Figure 6:**
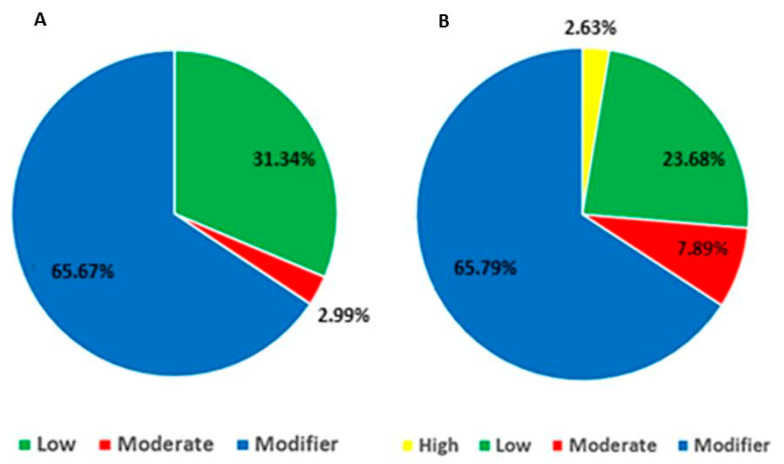
Impact caused by variants and its frequency. Legend: (**A**) samples from the scrotal hernia group. (**B**) Samples from the umbilical hernia group.

In the SH group, the genes corresponding to exonic regions, in which the variants were observed, were not enriched by the KEGG Pathway database [54]. Considering the type of impact caused by the variants, the results were distributed as shown in Figure 6A,B for the scrotal and umbilical hernias, respectively. These figures show that more than 60% of the variants represent variations of modifying impact for both types of hernia.

Among the transcripts present in the analyzed samples, five miRNAs were identified in the SH transcriptome and four in the UH transcriptome (Table 11). From these, three were expressed in both types of hernia. No DE miRNAs belonging to the evaluated samples were identified.

## 4. Discussion

Some studies investigating genes involved with the occurrence of hernias have been performed using candidate genes and GWAS approaches [11,25,29,31,32,57,58,59,60]. More recently, functional candidate genes were prospected for scrotal [6] and umbilical [7] hernias by our group using RNA-Seq approach. Nevertheless, since the molecular mechanisms involved with these anomalies are not yet completely understood, a comparison between the transcriptome of umbilical and scrotal hernias was performed here, allowing the identification of several common genes differentially expressed in both conditions. Moreover, several SNPs involved with these conditions were also identified and characterized.

### 4.1. Transcriptome Characterization

Gene expression studies obtained from samples of the inguinal and the umbilical ring tissue are quite recent. Lorenzetti et al. [5] and Romano et al. [6] performed gene expression analyses from the pig inguinal ring and Souza et al. [7] performed analyses with umbilical ring samples to investigate scrotal and umbilical hernias, respectively. Information about gene expression in those tissues is scarce and there are still many gaps to be elucidated in this field. Those authors found DE genes and pathways in each type of hernia. Since some genes and biological processes seem to be shared between SH and UH, new analyses were performed with focus on tissues characterization and on common processes involved with both types of hernia.

From the transcripts characterization of the two tissues (Table 1), a great similarity between the groups of both types of hernia was observed when comparing the number of each class of transcripts, implying that the appearance of both hernias may be related to the same set of genes or family of genes. This large number of transcripts that are expressed in both groups can also be seen in the Venn diagram (Figure 3). With the exception of processed pseudogenes and snRNA, which were not identified in the UH group, the percentage of each type of transcript was similar. Thus, the expression profile of the genes in the inguinal ring tissue was very similar to the profile found in the umbilical ring, being compatible with the histopathological composition of these two tissues (Figure 2).

### 4.2. Common Differentially Expressed Genes in Scrotal and Umbilical Hernias

From the DE genes observed in each type of hernia, 35 were common to both groups. Among these, nine genes (*CD2, GPT2, MOXD1*, ENSSSCG00000031037, ENSSSCG00000032582, ENSSSCG00000036224, ENSSSCG00000036983, ENSSSCG00000037009 and ENSSSCG00000039111) had different expression profiles when comparing both types of hernia. This behavior may have occurred due to the expression be tissue specific (inguinal ring and umbilical ring) for those genes. Other reasons could be the differences in sex and age between the groups evaluated for the two types of hernia. The other 26 DE genes have shown similar expression in both types of hernia, of which 14 genes were downregulated and 12 were upregulated in pigs affected by both types of hernia.

From the gene interaction network (Figure 5), three DE genes were enriched in both types of hernia. The *MAP1LC3C* (microtubule associated protein 1 light chain 3 γ) interacted in the group of the macroautophagy BP (GO: 0016236) [61]. Macroautophagy is the main path involved in inducing general renewal of cytoplasmic constituents in eukaryotic cells and is essential for cell survival, development, differentiation and homeostasis [62,63,64,65,66]. The Gene Ontology (GO) annotations related to this gene include the assembly and maturation of the autophagosome (Supplementary Materials 1: Table S4). Marcelino et al. [67] indicated the *MAP1LC3C* gene as a candidate for the formation of UH in pigs since this gene was upregulated in the affected compared to the normal pigs. In our research, the *MAP1LC3C* gene exhibited the same behavior, being upregulated in affected animals of both types of hernia when compared to the control groups (Figure 4A). Moreover, this expression profile can be one of the causes of the hernia onset, since the high expression of this gene can cause excessive autophagy and interfere with normal tissue development [68].

The *CFI* gene (Complement Factor I) was grouped in the cluster of the coagulation cascade metabolic pathway and complement system (Figure 5). The coagulation cascade is a sequence of interconnected reactions in order to clot the local blood when a blood vessel injury occurs [69]. The complement system is a proteolytic cascade in blood plasma and a mediator of innate immunity [70]. The GO annotations related to this gene include endocytosis (content absorption through membrane invagination process) and proteolysis (protein degradation process) (Supplementary Materials 1: Table S2). The CFI gene, like the *MAP1LC3C*, was upregulated in animals with hernia when compared to the control group (Figure 4A). The *CFI* encodes the trypsin-like protein serine-protease [42], which plays an essential role in regulating the immune response, controlling all the complement pathways [71]. The participation of the *CFI* in these pathways and processes, taken together with its expression profile, suggests that this gene could be involved with the consequence of these disorders.

The *MUC16* (Mucin-16) gene encodes a protein of the mucin family, which are O-glycosylated proteins found in the apical surfaces of the epithelium and play an important role in the formation of a protective mucous barrier [72]. This gene was enriched in the gene network (Figure 5) as a participant in processing O-glycan BP (GO: 16266) [61]. This process is related to the gradual addition of carbohydrate residues or carbohydrate derivatives to form the O-glycan structure [73]. The *MUC16* gene was also enriched as an integral cell membrane component BP (GO: 16021) [61]. According to Blalock et al. [74], the MUC16 build a protective barrier to the epithelial cell surface, where binding proteins are associated with its tail, linking it to the actin cytoskeleton. This gene was upregulated in the affected group of both types of hernia compared to their respective control groups (Figure 4A), thus configuring a defense system that might have arisen as a consequence of the hernias formation.

### 4.3. Enriched Biological Processes

When the 99 BP enriched by the 26 DE genes with an equivalent profile in both types of hernia (Supplementary Materials 1: Table S3) were evaluated, the BP of cell adhesion, apoptosis, organization of the actin cytoskeleton and organization of collagen fibrils can be highlighted, because they are generally linked to the formation of hernias [5,6,7]. The enriched genes for cell adhesion, *VIT* (Vitrin), *ACER2* (Alkaline Ceramidase 2) and *CHL1* (Cell Adhesion Molecule L1 Like) were downregulated in the affected animals compared to the control groups and *ACAN* (Aggrecan) was upregulated in the affected animals. The cell adhesion BP allows the interaction among cells, and between cells and the extracellular matrix [75]. This BP has already been related to tissue maintenance and cell differentiation [76,77]. The *CHL1* gene was enriched with the process of homophilic cell adhesion via plasma membrane adhesion molecules. *ACER2* participates in the specific BP of negative regulation of cell adhesion mediated by integrin and negative regulation of cell matrix adhesion. The *VIT* gene enriched the process of positive regulation of cell substrate adhesion. Thus, the reduced expression of these genes that actively participate in cell adhesion interferes with the integrity of tissues, which can be determinant for the appearance of both types of hernia.

The *KCNMA1* gene (Potassium Calcium—Activated Channel Subfamily M α 1), which was upregulated in animals affected with hernia, and *ACER2* (downregulated) were enriched in the apoptosis BP. This process is related to the regulation of programmed cell death, which is extremely important for the maintenance of the development of living beings [78]. The overexpression of the *KCNMA1* gene can compromise the tissue as a result of an accumulation of immature cells in the region, which can influence the appearance of hernias, especially when associated with unfavorable environmental factors. *ACER2* was enriched with the specific process of activating cysteine-type endopeptidase activity, involved in the apoptotic process, and the *KCNMA1* was enriched for positive regulation of the apoptotic process. This last gene was also enriched for the relaxation process of the vascular smooth muscle that is related to the negative regulation of the contraction of this muscle. The relaxation is mediated by a decrease in the phosphorylation state of the myosin light chain [79]. As the expression of this gene was higher in herniated than in normal pigs, the *KCNMA1* can be pointed out as a candidate gene for the formation of umbilical and scrotal hernia, since the lack of local muscle contraction facilitates the passage of the abdominal content through the rings.

Biological processes that regulate the activities of collagen and its structures have been indicated in the enrichment of the *ACAN* and *VIT* genes. The first gene was related to the condensation of mesenchymal cells that differentiate into chondrocytes, organization of collagen fibrils and the development of chondrocytes [61]. The *VIT* gene, on the other hand, was related to the morphogenesis of chondrocytes in the cartilage of the growth plate, in which the structures of a chondrocyte are generated and organized [80]. The *ACAN* gene was upregulated in animals affected with hernia, which is in accordance with the histopathological analyses that evidenced a larger amount of collagen compared to normal pigs. Moreover, *ACAN* upregulation in animals affected with hernia can generate an exaggerated collagen production, which has already been related to hernia previously [81].

Regarding the organization of the cytoskeleton, especially those processes related to actin, two genes were enriched, *SYNPO2* (Synaptopodin 2) and *ENSSSCG00000037142*. *SYNPO2* has been enriched specifically for the process of positive regulation of the actin filament bundles set. The organization of the actin cytoskeleton is carried out at the cellular level and results in the assembly, disposition of the constituent parts or disassembly of the structures, including filaments and their associated proteins [61]. In our study, this gene was downregulated in animals with hernia. This negative regulation can be a predisposing factor to hernia, since the non-assembly and organization of the structures that constitute the tissue can make it less resistant [18].

### 4.4. DE Genes Located in QTL Regions for Hernias and Polymorphisms Characterization

Several studies have been carried out to identify QTL regions related to umbilical and scrotal hernia [11,20,29,81,82]. Among the DE genes in the two types of hernia, *ACAN* and *BCHE* (Butyrylcholinesterase) are highlight since they have already been located in QTL regions associated to scrotal/inguinal hernia [20,29]. Even with scientific reports relating these two genes only with QTL regions for scrotal hernia, in our study, the expression profile of these two genes was equivalent in both types of hernia, being upregulated in the affected animals. Souza et al. [7] have recently indicated *ACAN* as a strong candidate gene for triggering umbilical hernias in pigs.

Our results have shown that variations in the transcripts may be related to the manifestation of the different types of hernia. In both groups, most of the polymorphisms detected were SNPs, followed by insertions and deletions (Supplementary Materials 1: Tables S4 and S5). In the SH group, a new SNP was identified on chromosome 13 (13: 34083960-34083960), which is located within a QTL region (QTL ID 55898) associated with scrotal hernia [11,20,29]. This SNP was mapped in the *PARP3* gene (member of the poly ADP-ribose 3 polymerase family), which acts in the repair pathways by base excision, apoptosis and necroptosis, participating in biological processes of DNA repair [4]. Moreover, Piórkowska et al. [83] carried out research with Polish Landrace and Pulawska pigs and pointed out the participation of the PARP3 gene in the regulation of the actin cytoskeleton BP. The muscle tissues belonging to the regions where the hernias occur are classified as skeletal striatum, which are formed by myofibrils composed by actin and myosin. As mentioned by Bendavid [18], disturbances in the structures of muscle fibers cause low resistance in the inguinal region, which can lead to scrotal hernia.

The 53 SNPs observed in the SH group were located in 17 genes (Additional file 1: Table S4), which have been enriched in nine biological processes (Table 9). Most of these BP were related to homeostasis, which are processes that maintain the stability of the structure of the analyzed tissue (GO:0042592) [61]. The *ACACA* gene, enriched in these BP, participates in processes that maintain the stability of anatomical structures of the site [43]. *ACACA* was downregulated in the SH-affected animals (Table 9) indicating the development of an unstable structure of the inguinal ring, which can influence the development of hernia. In humans, this gene participates in the fatty acid synthesis BP [43], which reinforces the histopathological findings that showed greater amount of adipose tissue in normal than in SH-affected pigs (Figure 2A,B). From the SNPs found in the SH group, all variants were tolerated (Table 7). According to the SIFT score, two had a moderate impact classification, so they can alter the effectiveness of the encoded protein. This means that the function of the proteins resulting from these sites has not been altered, since the SIFT score is a tool that predicts whether the variant affects the function of the protein or not [35]. These SNPs were located in two genes, *RAI14* and *RALGAPA1*; the first has already been annotated and the second has no identification in the VEP tool. No high impact polymorphisms were identified in the SH group.

The 67 SNPs found in the UH group were mapped in 24 genes (Supplementary Materials 1: Table S5). These genes were enriched in six biological processes (Table 10) [41], all of which were related to some type of regulation, mainly metabolic. The *EPHB2* and *VIM* genes were enriched in BP that interrupts the processes of cellular projections formation (GO: 0031345) [61]. These two genes were upregulated in animals affected with UH when compared to the control group (Table 10). The *VIM* gene encodes an intermediate filament protein that is part of the cytoskeleton [43]. Lazarides [84] reported that high amount of this filament is observed in the early stages of myogenesis in humans, and is hardly identified in adult muscles. Thus, the levels of this protein indicate functionality feature. The upregulation of *VIM* in the umbilical ring tissue of the UH-affected animals suggests that this gene may be involved with a consequence of UH since Miller et al. [12] reported that the appearance of hernia can be a consequence of a muscular defect.

Polymorphisms that had a high impact rating in the UH group (Supplementary Materials 1: Table S5) were identified in two genes (*NCOA7* and *SEC62*). These variants still do not have identification in the tool used, but they were classified as insertions of the Frameshift type. Therefore, they can cause an interruption in the translation reading frame, because the number of inserted nucleotides is not multiple of three [35]. The *NCOA7* (Nuclear receptor coactivator 7) is involved in the biological process of RNA polymerase II transcription and negative regulation of the cellular response to oxidative stress. The *SEC62* (Preprotein translocation factor) is related to the regulation of post-translational protein transport to the membrane BP and was mapped in a QTL region for stillborn pigs [85]. The detection of these polymorphisms is important because they can alter not only processes related to hernias, but all important processes for biological maintenance, possibly resulting in transcription failures or disruption in the transport of translated proteins by lack of regulation.

The SNPs classified as having moderate impact for the UH group were found in six genes (Supplementary Materials 1: Table S5), with the SNP rs327289001 being highlighted due to its deleterious SIFT score. This SNP is located in the *ITGAM* gene that participates in the biological process of ectodermal cell differentiation [35]. This process is related to the specialization of previously non-specialized cells, which acquire structure and functioning of ectodermic cells. This differentiation integrates the processes involved in the commitment of a cell to its specific purpose (GO: 0010668) [61]. In the embryonic gastrulation phase, the formation of germ layers (ectoderm, mesoderm and endoderm) occurs, which will give rise to specific tissues and organs [86]. The ectoderm is the external layer of a developing embryo and gives rise to epidermis, hair, nails, cutaneous and mammary glands, tooth enamel, inner ear, lens, and the anterior part of the pituitary gland, besides others related to the neural tube and neural crest [86]. A SNP with deleterious SIFT score indicates that the function of the protein can be altered due to the polymorphism, which in this case can result in non-differentiated cells, compromising the formation of resistant tissues, which, when associated with environmental factors such as obesity, can lead to hernia. SNPs located in QTL regions associated with UH were not found in the current study.

The SNP rs339972872 from the SH group and the SNPs rs324236192 and rs340781986 from the UH group were located in the same gene (*ACACA*) (Supplementary Materials 1: Tables S4 and S5). These are synonym SNPs and were classified as low impact. According to Stachowiak et al. [87], the *ACACA* gene is involved with performance traits in pigs.

From the expressed miRNAs, three were identified in the groups of both hernias. One of them, ssc-mir-214, plays an important role in the regulation of ovarian function and in the induction of granular ovarian cells to induce follicular development [88]. The ssc-mir-145, which was identified only in samples from the SH group, is involved in the development of adipose tissue [89].

We conducted the first comparative study of the pig inguinal and umbilical ring tissue transcriptomes. The results demonstrated similarities related to the expression profile of the whole transcriptome and DE genes in both types of hernia. The *ACAN* gene, which had already been associated to the appearance of scrotal hernia, showed similar behavior in the data obtained from the umbilical hernia group. Moreover, the *MAP1LC3C*, *VIT*, *ACER2*, *KCNMA1* and *SYNPO2* genes were highlighted as candidates for the formation of the two types of hernias evaluated in our studied for presenting equivalent expression in both hernias and for being involved in biological processes such as cell adhesion, cytoskeleton organization, collagen production, muscle relaxation and autophagy. Furthermore, the differential expression of some of those genes, such as *MAP1LC3C*, *VIT*, *ACER2* and *ACAN*, has already been confirmed using qPCR [6,7]. However, further studies are needed to identify the expression profile of these same genes in younger animals to improve our interpretation of the gene regulation mechanisms triggering the formation of hernias. The knowledge of the genetic factors that control the manifestation of both scrotal and umbilical hernia brings possibilities to the pig production chain to develop actions to reduce the appearance of these defects in their herds, aiming to reduce economical losses and favoring the animal welfare.

## 5. Conclusions

The expression profile of the inguinal and umbilical ring transcriptomes showed great similarity. Thirty-five differentially expressed genes between normal and affected samples were common to both types of hernia. The *MAP1LC3C*, *ACAN*, *VIT*, *ACER2*, *KCNMA1* and *SYNPO2* genes are indicated as strong candidates for the appearance of both defects. A total of 11 and 14 new SNPs were identified in the samples related to the scrotal hernia and umbilical hernia, respectively. Moreover, a SNP with predicted deleterious function was identified in the *ITGAM* gene, which might be related to the appearance of umbilical hernia in pigs. Finally, the expression profile of these genes possibly interferes with the normal development of the tissues, causing weakness and decreasing the resistance of the site, which can lead to the formation of both types of hernia in pigs.

## Figures and Tables

**Table 1:** Characterization of the transcripts identified in the inguinal and umbilical ring samples.

Annotated Transcripts	SH		UH	
LncRNA	68	0.53%	77	0.60%
MiRNA	5	0.04%	4	0.03%
Mt rRNA	2	0.02%	2	0.02%
Mt tRNA	1	0.01%	1	0.01%
Processed pseudogene	1	0.01%	0	0%
Protein-coding	12,601	98.55%	12,598	98.50%
Pseudogene	90	0.70%	91	0.71%
Ribozime	1	0.01%	1	0.01%
ScaRNA	1	0.01%	1	0.01%
SnoRNA	13	0.10%	14	0.11%
SnRNA	1	0.01%	0	0%
Y RNA	2	0.02%	1	0.01%
Total annotated transcripts	12,786		12,790	

**Table 2:** Differentially expressed genes identified in both scrotal (SH) and umbilical hernia (UH) groups.

ENSEMBL ID	Gene Symbol	Chromosome	Gene Name	SH-logFC	SH-FDR	UH-logFC	UH-FDR
ENSSSCG00000001832	*ACAN*	7	Aggrecan	2.913	0.001	2.788	0.040
ENSSSCG00000034213	*ACER2*	1	Alkaline ceramidase 2	−3.066	0.001	−2.373	0.004
ENSSSCG00000036223	*ACKR1*	4	Atypical chemokine receptor 1 (Duffy blood group)	−1.119	0.030	−1.023	0.034
ENSSSCG00000010370	*ANXA8*	14	Annexin A8	2.026	0.000	1.744	0.004
ENSSSCG00000032709	*ARL4A*	9	ADP ribosylation factor like GTPase 4A	−1.308	0.001	−1.199	0.031
ENSSSCG00000033350	*BCHE*	13	Butyrylcholinesterase	1.011	0.028	1.557	0.009
ENSSSCG00000028567	*BTNL9*	2	Butyrophilin like 9	−1.356	0.010	−2.268	0.016
ENSSSCG00000002662	*C16orf74*	6	Chromosome 16 open reading frame 74	1.818	0.025	1.661	0.008
ENSSSCG00000006736	*CD2*	4	CD2 molecule	2.275	0.028	−1.990	0.002
ENSSSCG00000009138	*CFI*	8	Complement factor I	2.254	0.000	1.904	0.029
ENSSSCG00000011524	*CHL1*	13	Cell adhesion molecule L1 like	−1.776	0.004	−1.321	0.009
ENSSSCG00000021588	*DAPK2*	1	Death associated protein kinase 2	−1.347	0.026	−1.473	0.013
ENSSSCG00000012126	*GPM6B*	X	Glycoprotein M6B	−1.047	0.009	−1.244	0.022
ENSSSCG00000002847	*GPT2*	6	Glutamic--pyruvic transaminase 2	−3.752	0.002	1.571	0.029
ENSSSCG00000036438	*GPX3*	16	Glutathione peroxidase 3	−2.184	0.000	−2.762	0.006
ENSSSCG00000017010	*INSYN2B*	16	Inhibitory synaptic factor family member 2B	3.436	0.000	1.667	0.020
ENSSSCG00000002245	*KATNBL1*	7	Katanin regulatory subunit B1 like 1	0.877	0.040	1.147	0.002
ENSSSCG00000010325	*KCNMA1*	14	Potassium calcium-activated channel subfamily M α 1	0.982	0.042	1.652	0.001
ENSSSCG00000034838	*MAP1LC3C*	10	Microtubule associated protein 1 light chain 3 γ	6.715	0.000	3.819	0.002
ENSSSCG00000004191	*MOXD1*	1	Monooxygenase DBH like 1	−2.980	0.010	2.570	0.033
ENSSSCG00000011133	*PFKFB3*	10	6-phosphofructo-2-kinase/fructose-2,6-biphosphatase 3	−1.135	0.008	−1.450	0.010
ENSSSCG00000007528	*PHACTR3*	17	Phosphatase and actin regulator 3	1.727	0.012	1.986	0.028
ENSSSCG00000007470	*RIPOR3*	17	RIPOR family member 3	−1.119	0.049	−1.377	0.042
ENSSSCG00000009111	*SYNPO2*	8	Synaptopodin 2	−1.293	0.006	−1.194	0.045
ENSSSCG00000014834	*UCP3*	9	Uncoupling protein 3	−2.693	0.024	−3.262	0.016
ENSSSCG00000008501	*VIT*	3	Vitrin	−2.317	0.004	−3.709	0.029
ENSSSCG00000015766	*WDR17*	15	WD repeat domain 17	−1.761	0.000	−1.773	0.019
ENSSSCG00000013714		2		2.087	0.048	1.813	0.019
ENSSSC00000037142		AEMK02000452.1	Cysteine-rich protein 1	1.222	0.000	−4.322	0.010
ENSSSCG00000031037		14		3.813	0.000	−4.021	0.029
ENSSSCG00000032582		14		3.873	0.000	−3.822	0.012
ENSSSCG00000036224		3		3.369	0.001	−3.010	0.045
ENSSSCG00000036983		AEMK02000452.1		4.568	0.001	−5.951	0.001
ENSSSCG00000037009		AEMK02000452.1		3.679	0.024	−3.785	0.031
ENSSSCG00000039111		AEMK02000452.1		4.596	0.000	1.180	0.041

**Table 3:** Differentially expressed genes in the inguinal and umbilical ring annotated as uncharacterized protein.

Gene ID	Description	e-Value	Query Cover (%)	Identity (%)	Accession (RefSeq)
ENSSSCG00000013714	Mucin-16 [*Sus scrofa*]	8 × 10^−157^	100	93.1	XP_020940777.1
ENSSSCG00000036224	Ig kappa chain V-C region (PLC18) [*Sus scrofa domesticus*]	5 × 10^−89^	82	85	PT0219
ENSSSCG00000031037	Immunoglobulin lambda-like polypeptide 5 precursor [*Sus scrofa*]	3 × 10^−71^	99	99.09	NP_001230248.1
ENSSSCG00000032582	Immunoglobulin lambda-like polypeptide 5 precursor [*Sus scrofa*]	7 × 10^−68^	94	92.04	NP_001230248.1
ENSSSCG00000036983	IgG heavy chain precursor [*Sus scrofa*]	0.0	74	79.78	BAM75547.1
ENSSSCG00000037009	IgG heavy chain precursor [*Sus scrofa*]	0.0	100	100	BAM75542.1
ENSSSCG00000039111	IgG heavy chain constant region [*Sus scrofa*]	1 × 10^−74^	100	100	BAM66306.1
ENSSSCG00000037142	Cysteine-rich protein 1 [*Camelus dromedarius*]	3 × 10^−41^	36	94.37	KAB1277051.1

**Table 4:** Macro biological processes (superclusters) enriched with the 35 differentially expressed genes common to both types of hernia.

Superclusters	Genes		
	Upregulated in Both Groups	Downregulated in Both Groups	Opposite Expression Profile
Cell adhesion (GO:0022610)		*CHL1*	*CD2*
Biological regulation (GO:0065007)	*ANXA8*	*ACKR1*, *SYNPO2*	*MOXD1*, ENSSSCG00000036224, ENSSSCG00000039111
Cellular process (GO:0009987)	*PHACTR3*, *ANXA8*, *MAP1LC3C*	*VIT, SYNPO2, BTNL9*	*GPT2*, *MOXD1*, *CD2*, ENSSSCG00000039111, ENSSSCG00000036224
Development (GO:0032502)	*PHACTR3*	*VIT*	
Immune system process (GO:0002376)		*ACER2*, *BTNL9*	*CD2*, ENSSSCG00000032582, ENSSSCG00000039111, ENSSSCG00000031037, ENSSSCG00000036224
Location (GO:0051179)	*ANXA8, MAP1LC3C*	*ARL4A*, *UCP3*	ENSSSCG00000036224, ENSSSCG00000039111
Metabolic process (GO:0008152)		*UCP3, PFKFB3*	*GPT2*, *MOXD1*, ENSSSCG00000036224, ENSSSCG00000039111
Multicellular organismal process (GO:0032501)	*ACAN, PHACTR3*	*ACKR1*, *CHL1*, *GPM6B, VIT*	
Response to stimulus (GO:0050896)		*ACKR1*, *GPX3, UCP3*	ENSSSCG00000037009, ENSSSCG00000036983, ENSSSCG00000036224, ENSSSCG00000039111

**Table 5:** Classification of polymorphisms found in samples from the inguinal and umbilical ring tissues from normal, and scrotal and umbilical hernia-affected pigs, respectively.

Polymorphism Type	Scrotal Hernia		Umbilical Hernia	
	N°	(%)	N°	(%)
Insertion	10	14.93	6	7.90
Deletion	4	5.97	3	5.26
SNP	53	79.10	67	86.84
Total	67	100	76	100

**Table 6:** Variants annotated in different functional classes in samples from inguinal and umbilical ring tissues.

Variant Type	Scrotal Hernia (%)	Umbilical Hernia (%)
Intronic	23.88	5.26
Synonym	29.85	23.68
Missense	2.99	7.89
Splicing	1.49	-
UTR5′	5.97	6.58
UTR3′	34.33	47.37
Downstream	1.49	6.58
Frameshift	-	2.63

**Table 7:** Missense variants observed in groups with sorting intolerant from tolerant (SIFT) score calculated in the dbSNP database (Ensembl).

Group	Variant	Location	Impact	Gene	SIFT
Scrotal hernia	rs325370594	16:20418972-20418972	Moderate	*RAI14*	Tolerant (1)
	-	7:64303141-64303141	Moderate	*RALGAPA1*	Tolerant (0.63)
Umbilical hernia	rs325089032	6:81571496-81571496	Moderate	*ELOA*	Tolerant (0.1)
	rs327289001	3:17254444-17254444	Moderate	*ITGAM*	Deleterious (0.01)
	rs789266896	3:17628688-17628688	Moderate	*RNF40*	Tolerant (0.6)
	rs330957838	3:17468302-17468302	Moderate	*SETD1A*	Tolerant low confidence (0.34)
	rs337670844	3:17399477-17399477	Moderate	*ZNF646*	Tolerant (0.08)
	rs323115420	3:16964045-16964045	Moderate	*ZNF713*	Tolerant (0.65)

**Table 8:** Genes with elevated impact variants enriched in metabolic pathways with the KEGG Pathway Database.

Variant	Gene	Pathway (ssc ¹)
New (Frameshift)	*SEC62*	Protein exports (ssc03060); Protein processing in the endoplasmic reticulum (ssc04141).
rs327289001 (Missense)	*ITGAM*	Rap1 signaling path (ssc04015); Phagosome (ssc04145); Cell adhesion molecules (CAMs) (ssc04514); Hematopoietic cell line (ssc04640); Transendothelial migration of leukocytes (ssc04670); Regulation of the actin cytoskeleton (ssc04810); Whooping cough (ssc05133); Legionellosis (ssc05134); Leishmaniasis (ssc05140); Amebiasis (ssc05146); Infection by Staphylococcus aureus (ssc05150); Tuberculosis (ssc05152); Incorrect regulation of transcription in cancer (ssc05202)
rs330957838 (Missense)	*SETD1A*	Lysine degradation (ssc00310)

¹ Metabolic pathway identifying code described for Sus scrofa by the KEGG Pathway.

**Table 9:** Biological processes enriched with genes harboring SNPs in the scrotal hernia group.

David Term	Biological Process	Enriched Genes
GO:0010604	Positive regulation of the metabolic process of macromolecules	*TBX3, **MYRF**, **MYLIP**, **PARP3***
GO:0055088	Lipid homeostasis	*ACACA, **MYYLIP***
GO:0009893	Positive regulation of the metabolic process	*TBX3, **MYRF, MYLIP, PARP3***
GO:0043170	Metabolic process of the macromolecule	*TBX3, **MYRF, MYLIP, PARP3**, ACACA, **DDB2***
GO:0065008	Regulation of biological quality	*TBX3, ACACA, **MYLIP, PARP3***
GO:0045935	Positive regulation of the compound metabolic process containing nucleobase	*TBX3, **MYRF, PARP3***
GO:0051173	Positive regulation of the metabolic process of nitrogen compounds	*TBX3, **MYRF, PARP3***
GO:0042592	Homeostatic process	*ACACA, **MYLIP, PARP3***
GO:0033554	Cellular stress response	*TBX3, **DDB2, PARP3***
GO:0060249	Anatomical structure homeostasis	*ACACA, **PARP3***

The genes in bold were upregulated in the scrotal hernia-affected group.

**Table 10:** Biological processes enriched with genes harboring single nucleotide polymorphisms (SNPs) in the umbilical hernia group.

GO Term	Biological Process	Enriched Genes
GO:0010468	Regulation of gene expression	*RPRD1A, RNF40, ELOA, **VIM**, ZNF629, **ZNF713***
GO:0010977	Negative regulation of the development of neuron projection	***EPHB2, VIM***
GO:0060255	Regulation of the metabolic process of macromolecules	***ITGB2**, RPRD1A, RNF40, ELOA, **VIM**, ZNF629, **ZNF713***
GO:0031345	Negative regulation of cellular projection organization	***EPHB2** e **VIM***
GO:0019222	Regulation of the metabolic process	***ITGB2**, RPRD1A, RNF40, ELOA, **VIM**, ZNF629, **ZNF713***
GO:0045665	Negative regulation of neuron differentiation	***EPHB2, VIM***

The genes in bold were upregulated in the umbilical hernia-affected group.

**Table 11:** miRNAs identified in the transcriptomes of the pig inguinal and umbilical ring tissues.

ENSEMBL ID	Name/Symbol	miRBase	Location	Group
ENSSSCG00000018513	ssc-mir-145 (MIR145)	MI0002417	2: 150.580.126-150.580.211	SH
ENSSSCG00000018758	ssc-mir-214 (MIR214)	MI0002441	9: 114.527.990-114.528.101	SH/UH
ENSSSCG00000019065	ssc-mir-186 (MIR186)	MI0002456	6: 141.943.328-141.943.409	SH
ENSSSCG00000034634	ssc-mir-6782	MI0031620	AEMK02000489.1: 40.305-40.379	UH
ENSSSCG00000035742	-	-	12: 14.578.144-14.578.210	SH/UH
ENSSSCG00000037094	ssc-mir-9810	MI0031577	4: 83.070.363-83.070.457	SH/UH

SH stands for scrotal hernia and UH for umbilical hernia.

## Data Availability

The datasets used or analyzed during the current study are available from the corresponding author on reasonable request. The transcriptome sequences for the scrotal and umbilical hernias are available in the SRA database with BioProject numbers PRJNA350530 and PRJNA445856, respectively. The SNP information is available in the EVA database with Project number PRJEB42670, Analyses number ERZ1737910.

## Supplementary Materials

The following are available online at https://www.mdpi.com/2073-4425/12/2/166/s1, Table S1: average of reads sequenced, removed in the quality control analysis and mapped in each group of samples, Table S2: biological processes of the 35 differentially expressed genes between normal and affected pigs common to both scrotal and umbilical hernias using DAVID database. Table S3: enrichment for biological process of the 26 DE genes with equivalent expression profile between both types of hernias using DAVID database. Table S4: polymorphisms identified in samples of the pig inguinal ring. Table S5: polymorphisms identified in samples of the pig umbilical ring. Figure S1: diagram summarizing the experiment and analyses performed. Figure S2: principal component analysis (PCA) plot S2A showing the separation of control (c) and affected (a) samples used to generate the transcriptome of the inguinal ring for scrotal hernia (SH), S2B showing the separation of control (c) and affected (a) samples used to generate the transcriptome of the umbilical ring for umbilical hernia (UH), and S2C with all samples together showing the separation of samples from both SH and UH transcriptomes.

## Abbreviations

BP	Biological process
DE	Differentially expressed
GATK	Genome analysis Tool Kit
GO	Gene ontology
HE	Hematoxylin and eosin
IH	Inguinal hernia
MDS	Multidimensional scaling
QTL	Quantitative trait loci
SH	Scrotal hernia
SIFT	Sorting intolerant from tolerant
SNP	Single nucleotide polymorphism
UH	Umbilical hernia
VEP	Variant effect predictor
