# Supplementary material for: Genes and SNPs Involved with Scrotal and Umbilical Hernia in Pigs
Source: Genes (Basel). 2021 Jan 27;12(2):166. doi: 10.3390/genes12020166 (PMC7912685; doi:10.3390/genes12020166)
Supplement: Supplementary file 1 [file genes-12-00166-s001.pdf]

Additional file 1

Table S1. Average of reads sequenced, removed in the quality control analysis and mapped in each group of samples.

**Samples**	Sequenced reads	Removed reads	Pairs reads	Mapped reads	% of mapped reads	Reads mapped in genes	% of reads mapped in genes
SH – N	28362296	5693664	22668632	21337739	94.10	17756382	83.22
SH – A	27838413	4232821	23605592	21850701	92.60	18446649	84.42
UH – N	24356034	2727661	21628373	19902246	91.99	16584959	83.33
UH – A	23738367	2214464	21523903	20502663	95.24	17607460	85.88

Legend: SH – N: Samples from the scrotal hernia group – normal pigs.
SH – A: Samples from the scrotal hernia group – affected pigs.
UH – N: Samples from the umbilical hernia group – normal pigs.
UH– A: Samples from the umbilical hernia group – affected pigs.

Table S2. Biological processes of the 35 differentially expressed genes between normal and affected pigs common to both scrotal and umbilical hernias using DAVID database.

GO Term	Bioprocesses	Enriched genes
GO:0001502	Cartilage condensation	***ACAN***
GO:0007155	Cell adhesion	***ACAN***, *CD2*
GO:0007417	Central nervous system development	***ACAN***
GO:0002063	Chondrocyte development	***ACAN***
GO:0030199	Collagen fibril organization	***ACAN***
GO:0007507	Heart development	***ACAN***
GO:0030166	Proteoglycan biosynthetic process	***ACAN***
GO:0001501	Skeletal system development	***ACAN***
GO:0009058	Biosynthetic process	*GPT2*
GO:0042420	Dopamine catabolic process	*MOXD1*
GO:0042421	Norepinephrine biosynthetic process	*MOXD1*
GO:0006589	Octopamine biosynthetic process	*MOXD1*
GO:0055114	Oxidation-reduction process	*MOXD1*, *GPX3*
GO:0032515	Negative regulation of phosphoprotein phosphatase activity	***PHACTR3***
GO:0043666	Regulation of phosphoprotein phosphatase activity	***PHACTR3***
GO:0030198	Extracellular matrix organization	*VIT*
GO:0003429	Growth plate cartilage chondrocyte morphogenesis	*VIT*
GO:0010811	Positive regulation of cell-substrate adhesion	*VIT*
GO:0032233	Positive regulation of actin filament bundle assembly	*SYNPO2*
GO:0006897	Endocytosis	***CFI***
GO:0006508	Proteolysis	***CFI***
GO:0030007	Cellular potassium ion homeostasis	***KCNMA1***
GO:0034220	Ion transmembrane transport	***KCNMA1***
GO:0006811	Ion transport	***KCNMA1***
GO:0060073	Micturition	***KCNMA1***
GO:0045794	Negative regulation of cell volume	***KCNMA1***
GO:0043065	Positive regulation of apoptotic process	***KCNMA1***
GO:1902632	Positive regulation of membrane hyperpolarization	***KCNMA1***
GO:0071805	Potassium ion transmembrane transport	***KCNMA1***
GO:0006813	Potassium ion transport	***KCNMA1***
GO:0034765	Regulation of ion transmembrane transport	***KCNMA1***
GO:0042391	Regulation of membrane potential	***KCNMA1***
GO:0060087	Relaxation of vascular smooth muscle	***KCNMA1***
GO:0051592	Response to calcium ion	***KCNMA1***
GO:0034465	Response to carbon monoxide	***KCNMA1***
GO:0001666	Response to hypoxia	***KCNMA1***
GO:0006970	Response to osmotic stress	***KCNMA1***
GO:0060083	Smooth muscle contraction involved in	***KCNMA1***

	micturition	
GO:0055085	Transmembrane transport	***KCNMA1***
GO:0016197	Endosomal transport	***ANXA8***
GO:0007032	Endosome organization	***ANXA8***
GO:1900138	Negative regulation of phospholipase A2 activity	***ANXA8***
GO:1900004	Negative regulation of serine-type endopeptidase activity	***ANXA8***
GO:0046835	Carbohydrate phosphorylation	*PFKFB3*
GO:0006003	Fructose 2,6-bisphosphate metabolic process	*PFKFB3*
GO:0006000	Fructose metabolic process	*PFKFB3*
GO:0016310	Phosphorylation	*PFKFB3, DAPK2*
GO:0008344	Adult locomotory behavior	*CHL1*
GO:0007411	Axon guidance	*CHL1*
GO:0050890	Cognition	*CHL1*
GO:0070593	Dendrite self-avoidance	*CHL1*
GO:0035640	Exploration behavior	*CHL1*
GO:0007156	Homophilic cell adhesion via plasma membrane adhesion molecules	*CHL1*
GO:0043524	Negative regulation of neuron apoptotic process	*CHL1*
GO:0001764	Neuron migration	*CHL1*
GO:0031175	Neuron projection development	*CHL1, GPM6B*
GO:0051612	Negative regulation of serotonin uptake	*GPM6B*
GO:1990845	Adaptive thermogenesis	*UCP3*
GO:1990542	Mitochondrial transmembrane transport	*UCP3*
GO:0006839	Mitochondrial transport	*UCP3*
GO:1902600	Proton transmembrane transport	*UCP3*
GO:0009409	Response to cold	*UCP3*
GO:0008150	Biological_process	*WDR17*
GO:0006468	Protein phosphorylation	*DAPK2*
GO:0050776	Regulation of immune response	*BTNL9*
GO:0050852	T cell receptor signaling pathway	*BTNL9*
GO:0006955	Immune response	*ENSSSCG00000031037, ENSSSCG00000036224*
GO:0002377	Immunoglobulin production	*ENSSSCG00000031037, ENSSSCG00000036224*
GO:0050873	Brown fat cell differentiation	*ARL4A*
GO:0006886	Intracellular protein transport	*ARL4A*
GO:0016192	Vesicle-mediated transport	*ARL4A*
GO:0006919	Activation of cysteine-type endopeptidase activity involved in apoptotic process	*ACER2*
GO:0006974	Cellular response to DNA damage stimulus	*ACER2*
GO:0035690	Cellular response to drug	*ACER2*
GO:0046514	Ceramide catabolic process	*ACER2*
GO:0006672	Ceramide metabolic process	*ACER2*
GO:0030330	DNA damage response, signal transduction by	*ACER2*

	p53 class mediator	
GO:0006629	Lipid metabolic process	*ACER2*
GO:0033629	Negative regulation of cell adhesion mediated by integrin	*ACER2*
GO:0001953	Negative regulation of cell-matrix adhesion	*ACER2*
GO:0090285	Negative regulation of protein glycosylation in Golgi	*ACER2*
GO:0010942	Positive regulation of cell death	*ACER2*
GO:0008284	Positive regulation of cell population proliferation	*ACER2*
GO:0042981	Regulation of apoptotic process	*ACER2*
GO:0010506	Regulation of autophagy	*ACER2*
GO:0032526	Response to retinoic acid	*ACER2*
GO:0046512	Sphingosine biosynthetic process	*ACER2*
GO:0035973	Aggrephagy	***MAP1LC3C***
GO:0000045	Autophagosome assembly	***MAP1LC3C***
GO:0097352	Autophagosome maturation	***MAP1LC3C***
GO:0006914	Autophagy	***MAP1LC3C***
GO:0000422	Autophagy of mitochondrion	***MAP1LC3C***
GO:0006995	Cellular response to nitrogen starvation	***MAP1LC3C***
GO:0009267	Cellular response to starvation	***MAP1LC3C***
GO:0016236	Macroautophagy	***MAP1LC3C***
GO:0070098	Chemokine-mediated signaling pathway	*ACKR1*
GO:0006954	Inflammatory response	*ACKR1*
GO:0032642	Regulation of chemokine production	*ACKR1*
GO:0006958	Complement activation, classical pathway	*ENSSSCG00000036224*
GO:0042742	Defense response to bacterium	*ENSSSCG00000036224*
GO:0006911	Phagocytosis, engulfment	*ENSSSCG00000036224*
GO:0006910	Phagocytosis, recognition	*ENSSSCG00000036224*
GO:0050871	Positive regulation of B cell activation	*ENSSSCG00000036224*
GO:0098869	Cellular oxidant detoxification	*GPX3*
GO:0042744	Hydrogen peroxide catabolic process	*GPX3*
GO:0051289	Protein homotetramerization	*GPX3*
GO:0006979	Response to oxidative stress	*GPX3*
GO:0030036	Actin cytoskeleton organization	***ENSSSCG00000037142***

*Genes in bold were upregulated in the affected group for both types of hernia, and those underlined were downregulated for one type of hernia and e upregulated for the other type of hernia.

Table S3. Enrichment for biological process of the 26 DE genes with equivalent expression profile between both types of hernias using DAVID database.

Ensembl ID	GO Term	Bioprocess
ENSSSCG00000037142	GO:0030036	Actin cytoskeleton organization
ENSSSCG00000034213	GO:0006919	Activation of cysteine-type endopeptidase activity involved in the apoptotic process
ENSSSCG00000014834	GO:1990845	Adaptive thermogenesis
ENSSSCG00000011524	GO:0008344	Adult locomotive behavior
ENSSSCG00000034838	GO:0035973	Aggrephagy
ENSSSCG00000034838	GO:0000045	Autophagosome assembly
ENSSSCG00000034838	GO:0097352	Autophagosome maturation
ENSSSCG00000034838	GO:0006914	Autophagy
ENSSSCG00000034838	GO:0000422	Mitochondria autophagy
ENSSSCG00000011524	GO:0007411	Axonal orientation
ENSSSCG00000015766	GO:0008150	Biological process
ENSSSCG00000032709	GO:0050873	Differentiation of brown fat cells
ENSSSCG00000011133	GO:0046835	Carbohydrate phosphorylation
ENSSSCG00000001832	GO:0001502	Condensation of cartilage
ENSSSCG00000001832	GO:0007155	Cell adhesion
ENSSSCG00000036438	GO:0098869	Cell oxidant detoxification
ENSSSCG00000010325	GO:0030007	Cell potassium ion homeostasis
ENSSSCG00000034213	GO:0006974	Cellular response to DNA damage stimulus
ENSSSCG00000034213	GO:0035690	Cellular response to the drug
ENSSSCG00000034838	GO:0006995	Cellular response to nitrogen starvation
ENSSSCG00000034838	GO:0009267	Cellular response to starvation
ENSSSCG00000001832	GO:0007417	Central nervous system development
ENSSSCG00000034213	GO:0046514	Catabolic ceramide process
ENSSSCG00000034213	GO:0006672	Metabolic process of ceramide
ENSSSCG00000036223	GO:0070098	Chemokine-mediated signaling pathway
ENSSSCG00000001832	GO:0002063	Chondrocyte development
ENSSSCG00000011524	GO:0050890	Cognition
ENSSSCG00000001832	GO:0030199	Organization of collagen fibrils
ENSSSCG00000011524	GO:0070593	Dendrite self-avoidance
ENSSSCG00000034213	GO:0030330	Response to DNA damage, signal transduction by the mediator of class p53
ENSSSCG00000009138	GO:0006897	Endocytosis
ENSSSCG00000010370	GO:0016197	Endosomal transport
ENSSSCG00000010370	GO:0007032	Endosomal organization
ENSSSCG00000011524	GO:0035640	Exploration behavior
ENSSSCG00000008501	GO:0030198	Organization of the extracellular matrix
ENSSSCG00000011133	GO:0006003	Metabolic process of fructose 2,6-bisphosphate
ENSSSCG00000011133	GO:0006000	Fructose metabolic process
ENSSSCG00000008501	GO:0003429	Morphogenesis of chondrocytes in the growth plate cartilage
ENSSSCG00000001832	GO:0007507	Heart development
ENSSSCG00000011524	GO:0007156	Homophilic cell adhesion via plasma membrane

		adhesion molecules
ENSSSCG00000036438	GO:0042744	Hydrogen peroxide catabolic process
ENSSSCG00000036223	GO:0006954	Inflammatory response
ENSSSCG00000032709	GO:0006886	Intracellular protein transport
ENSSSCG00000010325	GO:0034220	Ion transmembrane transport
ENSSSCG00000010325	GO:0006811	Ion transport
ENSSSCG00000034213	GO:0006629	Lipid metabolic process
ENSSSCG00000034838	GO:0016236	Macroautophagy
ENSSSCG00000010325	GO:0060073	Urination
ENSSSCG00000014834	GO:1990542	Mitochondrial transmembrane transport
ENSSSCG00000014834	GO:0006839	Mitochondrial transport
ENSSSCG00000034213	GO:0033629	Negative regulation of cell adhesion mediated by integrin
ENSSSCG00000010325	GO:0045794	Negative regulation of cell volume
ENSSSCG00000034213	GO:0001953	Negative regulation of cell matrix adhesion
ENSSSCG00000011524	GO:0043524	Negative regulation of the neuron apoptotic process
ENSSSCG00000010370	GO:1900138	Negative regulation of phospholipase A2 activity
ENSSSCG00000007528	GO:0032515	Negative regulation of phosphoprotein phosphatase activity
ENSSSCG00000034213	GO:0090285	Negative regulation of protein glycosylation in Golgi
ENSSSCG00000010370	GO:1900004	Negative regulation of serine endopeptidase activity
ENSSSCG00000012126	GO:0051612	Negative regulation of serotonin uptake
ENSSSCG00000011524	GO:0001764	Neuron migration
ENSSSCG00000011524	GO:0031175	Development of neuron projection
ENSSSCG00000012126	GO:0031175	Development of neuron projection
ENSSSCG00000036438	GO:0055114	Oxidation reduction process
ENSSSCG00000011133	GO:0016310	Phosphorylation
ENSSSCG00000021588	GO:0016310	Phosphorylation
ENSSSCG00000009111	GO:0032233	Positive regulation of the actin filament bundle assembly
ENSSSCG00000010325	GO:0043065	Positive regulation of the apoptotic process
ENSSSCG00000034213	GO:0010942	Positive regulation of cell death
ENSSSCG00000034213	GO:0008284	Positive regulation of cell population proliferation
ENSSSCG00000008501	GO:0010811	Positive regulation of cell substrate adhesion
ENSSSCG00000010325	GO:1902632	Positive regulation of membrane hyperpolarization
ENSSSCG00000010325	GO:0071805	Transmembrane transport of potassium ions
ENSSSCG00000010325	GO:0006813	Transport of potassium ions
ENSSSCG00000036438	GO:0051289	Protein homotetramerization
ENSSSCG00000021588	GO:0006468	Protein phosphorylation
ENSSSCG00000001832	GO:0030166	Proteoglycan biosynthetic process
ENSSSCG00000009138	GO:0006508	Proteolysis
ENSSSCG00000014834	GO:1902600	Transmembrane proton transport

ENSSSCG00000034213	GO:0042981	Regulation of the apoptotic process
ENSSSCG00000034213	GO:0010506	Autophagy regulation
ENSSSCG00000036223	GO:0032642	Regulation of chemokine production
ENSSSCG00000028567	GO:0050776	Regulation of the immune response
ENSSSCG00000010325	GO:0034765	Regulation of transmembrane ion transport
ENSSSCG00000010325	GO:0042391	Regulation of the membrane potential
ENSSSCG00000007528	GO:0043666	Regulation of phosphoprotein phosphatase activity
ENSSSCG00000010325	GO:0060087	Vascular smooth muscle relaxation
ENSSSCG00000010325	GO:0051592	Response to calcium ion
ENSSSCG00000010325	GO:0034465	Response to carbon monoxide
ENSSSCG00000014834	GO:0009409	Cold response
ENSSSCG00000010325	GO:0001666	Response to hypoxia
ENSSSCG00000010325	GO:0006970	Response to osmotic stress
ENSSSCG00000036438	GO:0006979	Response to oxidative stress
ENSSSCG00000034213	GO:0032526	Response to retinoic acid
ENSSSCG00000001832	GO:0001501	Development of the skeletal system
ENSSSCG00000010325	GO:0060083	Smooth muscle contraction involved in urination
ENSSSCG00000034213	GO:0046512	Sphingosine biosynthetic process
ENSSSCG00000028567	GO:0050852	T cell receptor signaling pathway
ENSSSCG00000010325	GO:0055085	Transmembrane transport
ENSSSCG00000032709	GO:0016192	Vesicle-mediated transport

Table S4. Polymorphisms identified in samples of the pig inguinal ring.

Variant	Polymorphism type	Location	Consequence	Impact	Symbol	Gene
New	Deletion	6:82851652-82851653	Intronic	Modifier	*RSRP1*	ENSSSCG00000034449
New	Deletion	7:104221878-104221879	UTR3'	Modifier	*SEL1L*	ENSSSCG00000002414
rs1108216348	Deletion	2:134533476-134533477	UTR5'	Modifier	*P4HA2*	ENSSSCG00000020915
rs1114124998	Deletion	5:9499095-9499096	Intronic	Modifier	*DDX17*	ENSSSCG00000000104
New	Insertion	13:122703207-122703207	UTR5'	Modifier	*VPS8*	ENSSSCG00000011788
New	Insertion	14:37596310-37596310	UTR3'	Modifier	*TBX3*	ENSSSCG00000009865
New	Insertion	2:101385604-101385604	UTR3'	Modifier	*MCTP1*	ENSSSCG00000025286
New	Insertion	7:104221039-104221039	UTR3'	Modifier	*SEL1L*	ENSSSCG00000002414
New	Insertion	7:104221369-104221369	UTR3'	Modifier	*SEL1L*	ENSSSCG00000002414
New	Insertion	7:104223100-104223100	UTR3'	Modifier	*SEL1L*	ENSSSCG00000002414
New	Insertion	8:99783036-99783036	UTR5'	Modifier	*ANKRD50*	ENSSSCG00000022173
rs698169541	Insertion	7:64368649-64368649	Intronic	Modifier	*RALGAPA1*	ENSSSCG00000032377
rs699814235	Insertion	1:45178864-45178864	UTR3'	Modifier	*PTP4A1*	ENSSSCG00000025941
rs707857689	Insertion	7:64370059-64370059	Intronic	Modifier	*RALGAPA1*	ENSSSCG00000032377
New	SNP	13:34083960-34083960	UTR3'	Modifier	*PARP3*	ENSSSCG00000023033
New	SNP	7:64303141-64303141	Missense	Modifier	*RALGAPA1*	ENSSSCG00000032377
rs318677442	SNP	2:134534068-134534068	Intronic	Modifier	*P4HA2*	ENSSSCG00000020915
rs319937494	SNP	7:12137039-12137039	UTR3'	Modifier	*MYLIP*	ENSSSCG00000001063
rs320772137	SNP	2:134568695-134568695	Intronic	Modifier	*PDLIM4*	ENSSSCG00000014274
rs320812009	SNP	7:64369632-64369632	Intronic	Modifier	*RALGAPA1*	ENSSSCG00000032377
rs321437076	SNP	2:15365561-15365561	Intronic	Modifier	*DDB2*	ENSSSCG00000013243
rs321448108	SNP	7:104223338-104223338	UTR3'	Modifier	*SEL1L*	ENSSSCG00000002414
rs321482827	SNP	16:20430280-20430280	Synonym	Low	*RAI14*	ENSSSCG00000016824
rs322028481	SNP	7:64370049-64370049	Downstream	Modifier	*INSM2*	ENSSSCG00000001949
rs323170636	SNP	2:134520258-134520258	Synonym	Low	*P4HA2*	ENSSSCG00000020915
rs323684157	SNP	7:104256746-104256746	Synonym	Low	*SEL1L*	ENSSSCG00000002414
rs324657636	SNP	2:134567763-	Intronic	Modifier	*P4HA2*	ENSSSCG00000020915

		134567763				
rs325370594	SNP	16:20418972-20418972	Missense	Modifier	*RAI14*	ENSSSCG00000016824
rs326057680	SNP	4:88876043-88876043	Intronic	Modifier	-	ENSSSCG00000006350
rs326292614	SNP	7:12136748-12136748	UTR3'	Modifier	*MYLIP*	ENSSSCG00000001063
rs327405343	SNP	2:134570264-134570264	UTR3'	Modifier	*PDLIM4*	ENSSSCG00000014274
rs328500299	SNP	2:134502458-134502458	UTR3'	Modifier	*P4HA2*	ENSSSCG00000020915
rs328617030	SNP	14:37595933-37595933	UTR3'	Modifier	*TBX3*	ENSSSCG00000009865
rs330964251	SNP	2:134569316-134569316	Splicing region Synonym	Low	*PDLIM4*	ENSSSCG00000014274
rs330986417	SNP	4:88559715-88559715	Synonym	Low	*OLFML2B*	ENSSSCG00000006345
rs331580151	SNP	2:134523104-134523104	Synonym	Low	*P4HA2*	ENSSSCG00000020915
rs331672044	SNP	7:104234431-104234431	Synonym	Low	*SEL1L*	ENSSSCG00000002414
rs331683704	SNP	7:12136979-12136979	UTR3'	Modifier	*MYLIP*	ENSSSCG00000001063
rs332730821	SNP	7:104239412-104239412	Synonym	Low	*SEL1L*	ENSSSCG00000002414
rs333120728	SNP	7:104256830-104256830	Synonym	Low	*SEL1L*	ENSSSCG00000002414
rs333503612	SNP	7:64368656-64368656	Intronic	Modifier	*RALGAPA1*	ENSSSCG00000032377
rs334410412	SNP	2:134520288-134520288	Synonym	Low	*P4HA2*	ENSSSCG00000020915
rs334902692	SNP	7:104247760-104247760	Synonym	Low	*SEL1L*	ENSSSCG00000002414
rs336367802	SNP	7:12133527-12133527	Synonym	Low	*MYLIP*	ENSSSCG00000001063
rs337177437	SNP	7:104254924-104254924	Synonym	Low	*SEL1L*	ENSSSCG00000002414
rs337356541	SNP	2:134566986-134566986	Intronic	Modifier	*PDLIM4*	ENSSSCG00000014274
rs337778431	SNP	2:134568657-134568657	Intronic	Modifier	*PDLIM4*	ENSSSCG00000014274
rs338426616	SNP	2:134570329-134570329	UTR3'	Modifier	*PDLIM4*	ENSSSCG00000014274
rs339297902	SNP	7:64369109-64369109	Intronic	Modifier	*RALGAPA1*	ENSSSCG00000032377
rs339523164	SNP	7:104223402-104223402	UTR3'	Modifier	*SEL1L*	ENSSSCG00000002414
rs339972872	SNP	12:38745989-38745989	Synonym	Low	*ACACA*	ENSSSCG00000017694
rs341675974	SNP	7:12136596-12136596	UTR3'	Modifier	*MYLIP*	ENSSSCG00000001063
rs341856430	SNP	7:64368731-64368731	Intronic	Modifier	*RALGAPA1*	ENSSSCG00000032377
rs342773249	SNP	7:64369687-64369687	Intronic	Modifier	*RALGAPA1*	ENSSSCG00000032377
rs343682724	SNP	4:88846962-88846962	UTR3'	Modifier	*FCRLA*	ENSSSCG00000030246
rs343732012	SNP	7:64372117-64372117	UTR3'	Modifier	*RALGAPA1*	ENSSSCG00000032377
rs344350584	SNP	2:134505397-134505397	Synonym	Low	*P4HA2*	ENSSSCG00000020915
rs345069964	SNP	6:74482956-	UTR5'	Modifier	*TMEM51*	ENSSSCG00000021026

		74482956				
rs345905406	SNP	7:104247817-104247817	Synonym	Low	*SEL1L*	ENSSSCG00000002414
rs346022448	SNP	2:134502393-134502393	UTR3'	Modifier	*P4HA2*	ENSSSCG00000020915
rs695824576	SNP	7:59713114-59713114	Synonym	Low	*CD276*	ENSSSCG00000001917
rs700364715	SNP	7:104221886-104221886	UTR3'	Modifier	*SEL1L*	ENSSSCG00000002414
rs712681004	SNP	2:9762085-9762085	UTR3'	Modifier	-	ENSSSCG00000013078
rs80801180	SNP	4:88559748-88559748	Synonym	Low	*OLFML2B*	ENSSSCG00000006345
rs81214013	SNP	2:134514074-134514074	Synonym	Low	*P4HA2*	ENSSSCG00000020915
rs81218171	SNP	2:134570121-134570121	Synonym	Low	*PDLIM4*	ENSSSCG00000014274
rs81365336	SNP	2:134516657-134516657	Synonym	Low	*P4HA2*	ENSSSCG00000020915

Table S5. Polymorphisms identified in samples of the pig umbilical ring.

Variant	Polymorphism type	Location	Consequence	Impact	Symbol	Gene
New	Deletion	3:16844063-16844064	UTR3'	Modifier	-	ENSSSCG00000020808
New	Deletion	3:17617319-17617323	UTR3'	Modifier	*ZNF629*	ENSSSCG00000007780
New	Deletion	6:80843072-80843073	UTR3'	Modifier	*EPHB2*	ENSSSCG00000003527
New	Deletion	6:80843074-80843081	UTR3'	Modifier	*EPHB2*	ENSSSCG00000003527
New	Insertion	1:37122230-37122230	Frameshift	High	*NCOA7*	ENSSSCG00000004222
New	Insertion	1:77585583-77585583	UTR3'	Modifier	*FYN*	ENSSSCG00000004421
New	Insertion	13:108676707-108676707	Frameshift	High	*SEC62*	ENSSSCG00000029608
New	Insertion	4:128903094-128903094	Intronic	Modifier	-	ENSSSCG00000047605
rs709055765	Insertion	6:82481037-82481037	UTR3'	Modifier	*CLIC4*	ENSSSCG00000038994
rs710955781	Insertion	13:50397619-50397619	UTR3'	Modifier	*EOGT*	ENSSSCG00000011504
New	SNP	12:3760896-3760896	UTR3'	Modifier	*AFMID*	ENSSSCG00000033636
New	SNP	13:207513656-207513656	UTR3'	Modifier	*ITGB2*	ENSSSCG00000025133
New	SNP	3:16078557-16078557	Synonym	Low	-	ENSSSCG00000007733
New	SNP	3:17082026-17082026	UTR3'	Modifier	*C16orf58*	ENSSSCG00000007753
New	SNP	3:17591144-17591144	Downstream	Modifier	*BCL7C*	ENSSSCG00000007776
New	SNP	3:51803551-51803551	UTR3'	Modifier	*IL18R1*	ENSSSCG00000008159
rs1107804156	SNP	3:17618533-17618533	Intronic	Modifier	*ZNF629*	ENSSSCG00000007780
rs1108762720	SNP	3:16851119-16851119	Synonym	Low	-	ENSSSCG00000020808
rs320729536	SNP	3:16382619-16382619	UTR3'	Modifier	*KCTD7*	ENSSSCG00000040985
rs322669402	SNP	3:17459844-17459844	UTR3'	Modifier	*HSD3B7*	ENSSSCG00000032369
rs323015047	SNP	6:119763116-119763116	UTR3'	Modifier	*RPRD1A*	ENSSSCG00000027700
rs323115420	SNP	3:16964045-16964045	Missense	Modifier	*ZNF713*	ENSSSCG00000029029
rs323662654	SNP	3:16384658-16384658	UTR3'	Modifier	*KCTD7*	ENSSSCG00000040985
rs323726488	SNP	3:16383246-16383246	UTR3'	Modifier	*KCTD7*	ENSSSCG00000040985
rs324198007	SNP	3:17491763-17491763	Synonym	Low	*ORAI3*	ENSSSCG00000007770
rs324205762	SNP	3:16971143-16971143	UTR5'	Modifier	*ZNF713*	ENSSSCG00000029029

rs324236192	SNP	12:38624714-38624714	Synonym	Low	*ACACA*	ENSSSCG00000017694
rs324583382	SNP	3:16384043-16384043	UTR3'	Modifier	*KCTD7*	ENSSSCG00000040985
rs325089032	SNP	6:81571496-81571496	Missense	Modifier	*ELOA*	ENSSSCG00000025440
rs325937498	SNP	3:17466691-17466691	Synonym	Low	*SETD1A*	ENSSSCG00000007782
rs326053487	SNP	3:16383454-16383454	UTR3'	Modifier	*KCTD7*	ENSSSCG00000040985
rs326115442	SNP	6:80824416-80824416	Synonym	Low	*EPHB2*	ENSSSCG00000003527
rs326942919	SNP	3:17246305-17246305	Synonym	Low	*ITGAM*	ENSSSCG00000007754
rs327289001	SNP	3:17254444-17254444	Missense	Modifier	*ITGAM*	ENSSSCG00000007754
rs327572607	SNP	6:80837841-80837841	Synonym	Low	*EPHB2*	ENSSSCG00000003527
rs327947675	SNP	3:17399455-17399455	Synonym	Low	*ZNF646*	ENSSSCG00000026817
rs329707669	SNP	3:16383919-16383919	UTR3'	Modifier	*KCTD7*	ENSSSCG00000040985
rs330195537	SNP	10:43520964-43520964	Intronic	Modifier	*VIM*	ENSSSCG00000011033
rs330731365	SNP	3:16844265-16844265	UTR3'	Modifier	-	ENSSSCG00000020808
rs330957838	SNP	3:17468302-17468302	Missense	Modifier	*SETD1A*	ENSSSCG00000007782
rs331463738	SNP	12:38129509-38129509	UTR3'	Modifier	*DHRS11*	ENSSSCG00000017690
rs332268785	SNP	3:16385727-16385727	UTR3'	Modifier	*KCTD7*	ENSSSCG00000040985
rs333208968	SNP	3:16383250-16383250	UTR3'	Modifier	*KCTD7*	ENSSSCG00000040985
rs333661817	SNP	3:16383284-16383284	UTR3'	Modifier	*KCTD7*	ENSSSCG00000040985
rs333780109	SNP	3:16971156-16971156	UTR5'	Modifier	*ZNF713*	ENSSSCG00000029029
rs334463568	SNP	18:11660822-11660822	UTR3'	Modifier	*CREB3L2*	ENSSSCG00000016520
rs335540465	SNP	3:17613531-17613531	Synonym	Low	*ZNF629*	ENSSSCG00000007780
rs336364520	SNP	3:16383470-16383470	UTR3'	Modifier	*KCTD7*	ENSSSCG00000040985
rs337670844	SNP	3:17399477-17399477	Missense	Modifier	*ZNF646*	ENSSSCG00000026817
rs339276563	SNP	3:17468216-17468216	Synonym	Low	*SETD1A*	ENSSSCG00000007782
rs339771716	SNP	3:17461694-17461694	Synonym	Low	*HSD3B7*	ENSSSCG00000032369
rs339771716	SNP	3:17461694-17461694	Downstream	Modifier	*STX1B*	ENSSSCG00000021238
rs340480028	SNP	3:17460060-17460060	UTR3'	Modifier	*HSD3B7*	ENSSSCG00000032369
rs340781986	SNP	12:38624687-38624687	Synonym	Low	*ACACA*	ENSSSCG00000017694
rs341960076	SNP	3:16855237-16855237	Synonym	Low	-	ENSSSCG00000020808
rs342012840	SNP	3:16971089-16971089	UTR5'	Modifier	*ZNF713*	ENSSSCG00000029029
rs342283188	SNP	6:80843098-80843098	UTR3'	Modifier	*EPHB2*	ENSSSCG00000003527

rs343615406	SNP	3:16971169-16971169	UTR5'	Modifier	*ZNF713*	ENSSSCG00000029029
rs343894209	SNP	3:17466745-17466745	Synonym	Low	*SETD1A*	ENSSSCG00000007782
rs343913735	SNP	3:16844271-16844271	UTR3'	Modifier	-	ENSSSCG00000020808
rs344858642	SNP	3:17244108-17244108	Synonym	Low	*ITGAM*	ENSSSCG00000007754
rs344892486	SNP	3:17610468-17610468	UTR5'	Modifier	*ZNF629*	ENSSSCG00000007780
rs345204099	SNP	3:16384270-16384270	UTR3'	Modifier	*KCTD7*	ENSSSCG00000040985
rs345481021	SNP	6:80824380-80824380	Synonym	Low	*EPHB2*	ENSSSCG00000003527
rs345676220	SNP	3:17491423-17491423	UTR3'	Modifier	*ORAI3*	ENSSSCG00000007770
rs346223430	SNP	6:80843336-80843336	UTR3'	Modifier	*EPHB2*	ENSSSCG00000003527
rs694561033	SNP	3:16385959-16385959	UTR3'	Modifier	*KCTD7*	ENSSSCG00000040985
rs697381317	SNP	3:17591079-17591079	Downstream	Modifier	*BCL7C*	ENSSSCG00000007776
rs699677308	SNP	3:17591133-17591133	Downstream	Modifier	*BCL7C*	ENSSSCG00000007776
rs701844432	SNP	3:16383956-16383956	UTR3'	Modifier	*KCTD7*	ENSSSCG00000040985
rs706065940	SNP	3:17591138-17591138	Downstream	Modifier	*BCL7C*	ENSSSCG00000007776
rs713060696	SNP	3:16383936-16383936	UTR3'	Modifier	*KCTD7*	ENSSSCG00000040985
rs713239492	SNP	3:17612980-17612980	Synonym	Low	*ZNF629*	ENSSSCG00000007780
rs789266896	SNP	3:17628688-17628688	Missense	Modifier	*RNF40*	ENSSSCG00000007786
rs793318116	SNP	3:17460656-17460656	Synonym	Low	*HSD3B7*	ENSSSCG00000032369
rs81389091	SNP	6:80842615-80842615	UTR3'	Modifier	*EPHB2*	ENSSSCG00000003527

Additional file 2

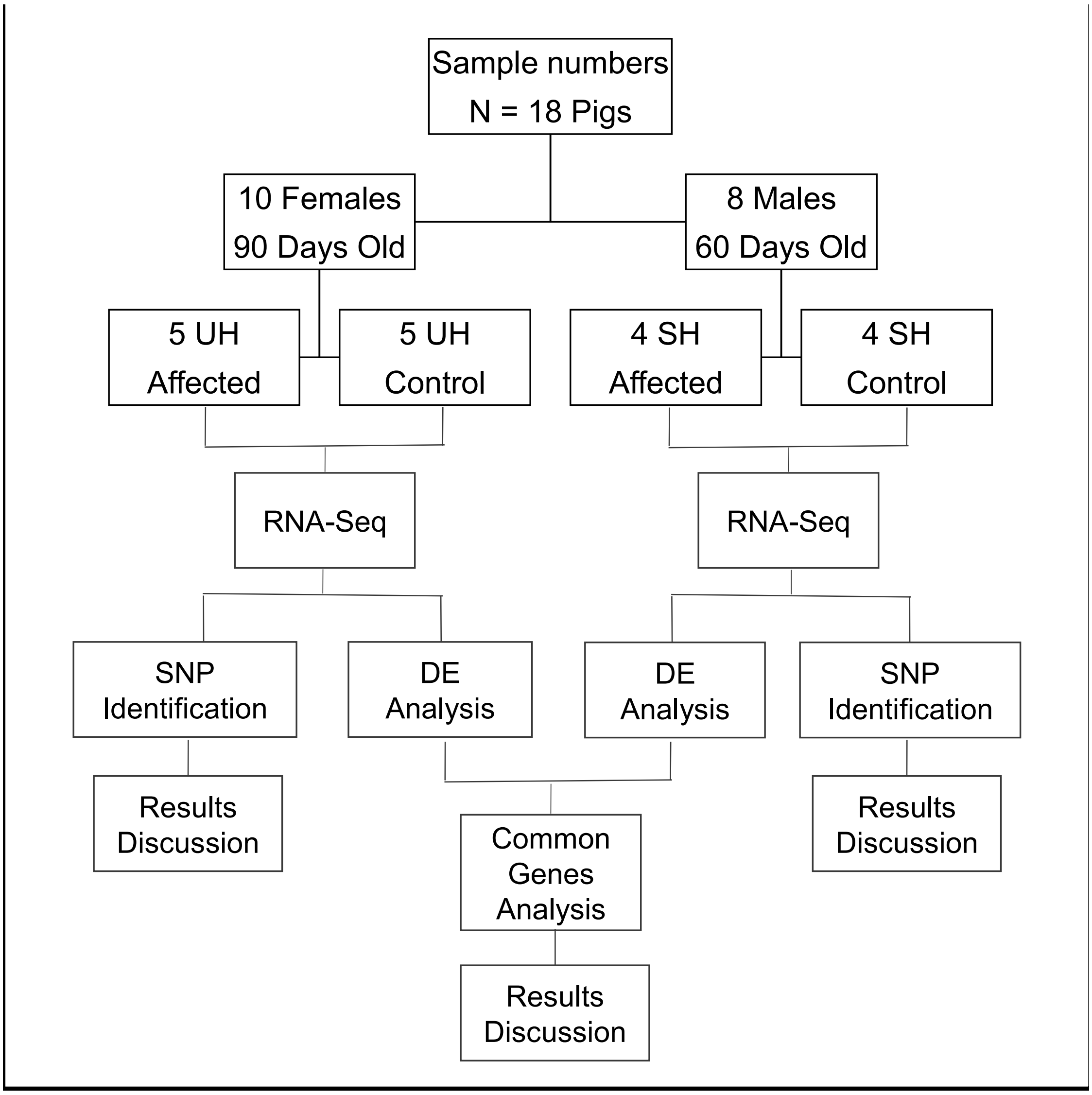

Figure S1. Diagram summarizing the experiment and analyses performed.

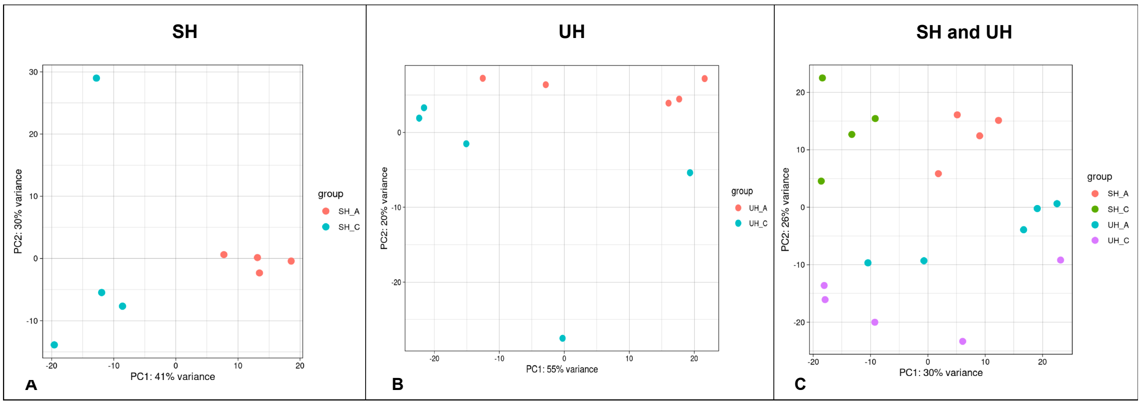

Figure S2. Principal component analysis (PCA) plot S2A showing the separation of control (c) and affected (a) samples used to generate the transcriptome of the inguinal ring for scrotal hernia (SH), S2B showing the separation of control (c) and affected (a) samples used to generate the transcriptome of the umbilical ring for umbilical hernia (UH), and S2C with all samples together showing the separation of samples from both SH and UH transcriptomes.
